# Correction: From displacement to hunger: How migration due to conflict affects food security in Yemen

**DOI:** 10.1371/journal.pone.0352770

**Published:** 2026-06-26

**Authors:** Sayed Jubair Bin Hossain, Niaz Makhdum, Maruf Hasan Rumi

The acronym IRR appears incorrectly throughout the article. The correct acronym is PRR.
