# Supplementary material for: From displacement to hunger: How migration due to conflict affects food security in Yemen
Source: PLoS One. 2025 Oct 14;20(10):e0333830. doi: 10.1371/journal.pone.0333830 (PMC12520372; doi:10.1371/journal.pone.0333830)
Supplement: Appendix Table 1 — (DOCX) [file pone.0333830.s001.docx]

# Supporting

| **Appendix Table 1** | | |
| --- | --- | --- |
| **Post-estimation Tests** | | |
|  |  |  |
| AIC and BIC to test goodness of fit | | |
| Hunger | AIC | BIC |
| Full model | 22961.06 | 23217.39 |
| Model without Migration Indicators | 22998.11 | 23216.74 |
|  |  |  |
| Without Eating | AIC | BIC |
| Full model | 12819.08 | 13075.41 |
| Model without Migration Indicators | 12877.95 | 13096.58 |
|  |  |  |
| Likelihood Ratio Test for goodness of fit | | |
| Migration Indicators Significantly Explain the Model of Hunger | LR Chi 2 = 47.05 | |
|  | p-value < 0.001 | |
|  |  |  |
| Migration Indicators Significantly Explain the Model of Without Eating | LR Chi2 = 68.87 | |
|  | p-value < 0.001 | |

Akaike Information criterion and Bayesian Information criterion are measures to assess the goodness of fit for the regression model. Lower levels of AIC and BIC represent a better fit for the model. AIC attempts to balance model fit and complexity by penalizing models with more parameters. BIC imposes a stronger penalty for the number of parameters than AIC. If both values are lower, that represents a better fit. However, if there are two different lower values, this study chooses AIC as it is more generally focused on goodness of fit (40) For this study, the full model, including all the migration variables, had a lower AIC value; therefore, the indicators explain both models of food insecurity better.

Another goodness-of-fit test used was the Likelihood Ratio test. This test determines whether the full model, which includes the migration indicators, statistically significantly explains food insecurity better than the reduced model (41). The Likelihood ratio test robustly shows that migration indicators explain the model better for both regression models.

**References:**

40. Burnham KP, Anderson DR. Multimodel Inference: Understanding AIC and BIC in Model Selection. Sociological Methods & Research. 2004 Nov 1;33(2):261–304.

41. Perneger TV. How to use likelihood ratios to interpret evidence from randomized trials. Journal of Clinical Epidemiology. 2021 Aug 1;136:235–42.
