# Supplementary material for: From displacement to hunger: How migration due to conflict affects food security in Yemen
Source: PLoS One. 2025 Oct 14;20(10):e0333830. doi: 10.1371/journal.pone.0333830 (PMC12520372; doi:10.1371/journal.pone.0333830)
Supplement: Attachment — Submitted filename: PONE-D-25-26248_R1.pdf [file pone.0333830.s004.pdf]

PLOS ONE

From Displacement to Hunger: How Migration Due to Conflict Affects Food Security in Yemen

--Manuscript Draft--

**Manuscript Number:**	PONE-D-25-26248R1
**Article Type:**	Research Article
**Full Title:**	From Displacement to Hunger: How Migration Due to Conflict Affects Food Security in Yemen
**Short Title:**	How Migration Due to Conflict Affects Food Security in Yemen
**Corresponding Author:**	Maruf Hasan Rumi, MSS<br>University of Dhaka<br>Dhaka, Dhaka BANGLADESH
**Keywords:**	Displacement; Hunger; migration; conflict; food security; Yemen
**Abstract:**	Background: Conflict-induced migration is a key driver of global food insecurity. Over the past decade, prolonged conflicts have displaced millions of people. This crisis plunged millions into acute hunger, deepening existing vulnerabilities and severely restricting access to food and livelihoods across Yemen.<br>Objective: This study aims to investigate the relationship between conflict-induced migration and food insecurity in Yemen, focusing on how displacement exacerbates hunger and malnutrition among affected populations.<br>Methodology: This study analyzed responses from 13,891 individuals across 22 governorates, using data from the Yemen Multiple Indicator Cluster Survey (MICS) 2022–2023. The two primary indicators of food insecurity — specifically, hunger frequency and instances of not eating for a full day — were examined. Additionally, Negative Binomial Regression was employed to model the over-dispersed count data while controlling for factors such as gender, age, education, household size, urban-rural location, and wealth.<br>Results: The findings revealed that approximately 24% of respondents experienced hunger, while about 12% went without food for an entire day. Moreover, conflict-induced migration substantially augmented the incidence of hunger (IRR = 1.262; 95% CI: 1.111–1.435) and going without eating for an entire day (IRR= 1.251; 95% CI: 1.032-1.516), though international migration to Yemen decreased food insecurity. Additionally, being female, having higher education, and belonging to wealthier households were protective factors against food insecurity, while urban residence significantly increased the likelihood of hunger (IRR = 2.231; 95% CI: 1.990- 2.501) and meal skipping (IRR = 3.657; 95% CI: 3.086-4.335).<br>Conclusion: Conflict-induced displacement is associated with worsened food insecurity, escalating hunger, and deprivation among displaced people, underscoring the immediate necessity of targeted humanitarian interventions in Yemen. Policy measures must prioritize the re-establishment of food systems and livelihoods in affected regions to alleviate the intensifying hunger crisis.
**Order of Authors:**	Sayed Jubair Bin Hossain
	Niaz Makhdum
	Maruf Hasan Rumi, MSS
**Response to Reviewers:**	I have revised the file accordingly to the reviewers and editor comment. If any further revision is needed, I am available to do so.
**Additional Information:**	
**Question**	**Response**
**Financial Disclosure**<br><br>Enter a financial disclosure statement that describes the sources of funding for the	The author(s) received no specific funding for this work.

Powered by Editorial Manager® and ProduXion Manager® from Aries Systems Corporation

work included in this submission. Review the submission guidelines for detailed requirements. View published research articles from *PLOS ONE* for specific examples.<br><br>This statement is required for submission and **will appear in the published article** if the submission is accepted. Please make sure it is accurate.<br><br>**Funded studies**<br>Enter a statement with the following details:<br>• Initials of the authors who received each award<br>• Grant numbers awarded to each author<br>• The full name of each funder<br>• URL of each funder website<br>• Did the sponsors or funders play any role in the study design, data collection and analysis, decision to publish, or preparation of the manuscript?<br><br>Did you receive funding for this work?	
**Competing Interests**<br><br>Use the instructions below to enter a competing interest statement for this submission. On behalf of all authors, disclose any competing interests that could be perceived to bias this work—acknowledging all financial support and any other relevant financial or non-financial competing interests.<br><br>This statement is required for submission and **will appear in the published article** if the submission is accepted. Please make sure it is accurate and that any funding sources listed in your Funding Information later in the submission form are also declared in your Financial Disclosure statement.<br><br>View published research articles from *PLOS ONE* for specific examples.	The authors have declared that no competing interests exist.

Powered by Editorial Manager® and ProduXion Manager® from Aries Systems Corporation

**NO authors have competing interests**<br><br>Enter: *The authors have declared that no competing interests exist.*<br><br>**Authors with competing interests**<br><br>Enter competing interest details beginning with this statement:<br><br>*I have read the journal's policy and the authors of this manuscript have the following competing interests: [insert competing interests here]*<br><br>* typeset	
**Ethics Statement**<br><br>Enter an ethics statement for this submission. This statement is required if the study involved:<br><br>• Human participants<br>• Human specimens or tissue<br>• Vertebrate animals or cephalopods<br>• Vertebrate embryos or tissues<br>• Field research<br><br>Write "N/A" if the submission does not require an ethics statement.<br><br>General guidance is provided below. Consult the submission guidelines for detailed instructions. **Make sure that all information entered here is included in the Methods section of the manuscript.**	N/A

**Format for specific study types**<br><br>**Human Subject Research (involving human participants and/or tissue)**<br>• Give the name of the institutional review board or ethics committee that approved the study<br>• Include the approval number and/or a statement indicating approval of this research<br>• Indicate the form of consent obtained (written/oral) or the reason that consent was not obtained (e.g. the data were analyzed anonymously)<br><br>**Animal Research (involving vertebrate animals, embryos or tissues)**<br>• Provide the name of the Institutional Animal Care and Use Committee (IACUC) or other relevant ethics board that reviewed the study protocol, and indicate whether they approved this research or granted a formal waiver of ethical approval<br>• Include an approval number if one was obtained<br>• If the study involved *non-human primates*, add *additional details* about animal welfare and steps taken to ameliorate suffering<br>• If anesthesia, euthanasia, or any kind of animal sacrifice is part of the study, include briefly which substances and/or methods were applied<br><br>**Field Research**<br><br>Include the following details if this study involves the collection of plant, animal, or other materials from a natural setting:<br>• Field permit number<br>• Name of the institution or relevant body that granted permission	
**Data Availability**<br><br>Authors are required to make all data underlying the findings described fully available, without restriction, and from the time of publication. PLOS allows rare exceptions to address legal and ethical concerns. See the PLOS Data Policy and FAQ for detailed information.	Yes - all data are fully available without restriction

A Data Availability Statement describing where the data can be found is required at submission. Your answers to this question constitute the Data Availability Statement and **will be published in the article**, if accepted.<br><br>**Important:** Stating 'data available on request from the author' is not sufficient. If your data are only available upon request, select 'No' for the first question and explain your exceptional situation in the text box.<br><br>Do the authors confirm that all data underlying the findings described in their manuscript are fully available without restriction?	
**Describe where the data may be found in full sentences. If you are copying our sample text, replace any instances of XXX with the appropriate details.**<br><br>• If the data are **held or will be held in a public repository**, include URLs, accession numbers or DOIs. If this information will only be available after acceptance, indicate this by ticking the box below. For example: *All XXX files are available from the XXX database (accession number(s) XXX, XXX.).*<br>• If the data are all contained **within the manuscript and/or Supporting Information files**, enter the following: *All relevant data are within the manuscript and its Supporting Information files.*<br>• If neither of these applies but you are able to provide **details of access elsewhere**, with or without limitations, please do so. For example:<br><br>*Data cannot be shared publicly because of [XXX]. Data are available from the XXX Institutional Data Access / Ethics Committee (contact via XXX) for researchers who meet the criteria for access to confidential data.*<br><br>*The data underlying the results presented in the study are available from (include the name of the third party*	https://ghdx.healthdata.org/record/yemen-multiple-indicator-cluster-survey-2022-2023

and contact information or URL).<br>• This text is appropriate if the data are owned by a third party and authors do not have permission to share the data.<br><br>* typeset	
Additional data availability information:	Tick here if the URLs/accession numbers/DOIs will be available only after acceptance of the manuscript for publication so that we can ensure their inclusion before publication.

Powered by Editorial Manager® and ProduXion Manager® from Aries Systems Corporation

Manuscript
Click here to access/download;Manuscript;Revised Manuscript (1).docx

From Displacement to Hunger: How Migration Due to Conflict Affects Food Security in Yemen

Sayed Jubair Bin Hossain[1]¶

Niaz Makhdum[2]¶

Maruf Hasan Rumi[3]*¶

[1] Department of Economics, University of Dhaka, Dhaka, Bangladesh.
Email: sjb.hossain@du.ac.bd; Orcid: https://orcid.org/0009-0001-3599-5293
[2] Department of Public Administration, Begum Rokeya University, Rangpur, Bangladesh.
Email: niaz.makhdum1995@gmail.com ; Orcid: https://orcid.org/0000-0003-0885-605X
[3] Department of Public Administration, University of Dhaka, Dhaka, Bangladesh.
Email: marufhasanrumi@du.ac.bd; Orcid: https://orcid.org/0000-0003-1752-5615

***Corresponding author**
Email: marufhasanrumi@du.ac.bd

From Displacement to Hunger: How Migration Due to Conflict Affects Food Security in Yemen

Abstract

Background: Conflict-induced migration is a key driver of global food insecurity. Over the past decade, prolonged conflicts have displaced millions of people. This crisis plunged millions into acute hunger, deepening existing vulnerabilities and severely restricting access to food and livelihoods across Yemen.

Objective: This study aims to investigate the relationship between conflict-induced migration and food insecurity in Yemen, focusing on how displacement exacerbates hunger and malnutrition among affected populations.

Methodology: This study analyzed responses from 13,891 individuals across 22 governorates, using data from the Yemen Multiple Indicator Cluster Survey (MICS) 2022–2023. The two primary indicators of food insecurity — specifically, hunger frequency and instances of not eating for a full day — were examined. Additionally, Negative Binomial Regression was employed to model the over-dispersed count data while controlling for factors such as gender, age, education, household size, urban-rural location, and wealth.

Results: The findings revealed that approximately 24% of respondents experienced hunger, while about 12% went without food for an entire day. Moreover, conflict-induced migration substantially augmented the incidence of hunger (IRR = 1.262; 95% CI: 1.111–1.435) and going without eating for an entire day (IRR= 1.251; 95% CI: 1.032-1.516), though international migration to Yemen decreased food insecurity. Additionally, being female, having higher education, and belonging to wealthier households were protective factors against food insecurity, while urban residence

significantly increased the likelihood of hunger (IRR = 2.231; 95% CI: 1.990- 2.501) and meal skipping (IRR = 3.657; 95% CI: 3.086-4.335).

Conclusion: Conflict-induced displacement is associated with worsened food insecurity, escalating hunger, and deprivation among displaced people, underscoring the immediate necessity of targeted humanitarian interventions in Yemen. Policy measures must prioritize the re-establishment of food systems and livelihoods in affected regions to alleviate the intensifying hunger crisis.

Keywords: *Displacement, Hunger, Migration, Conflict, Food Security, Yemen.*

Introduction

Migration caused by conflict has become a leading global crisis of the 21st century, with severe socioeconomic and humanitarian effects. The armed conflict in Yemen, ongoing since 2014, has resulted in the displacement of millions of people, both internally and externally (1). The total number of people living in internal displacement increased by 51% over the past five years, reaching a record high of 75.9 million people across 116 countries at the end of 2023. Of the 75.9 million Internally Displaced People (IDPs) at the end of 2023 globally, 68.3 million were displaced by conflict and violence, and in Yemen, this number is 4.5 million (2). Forced displacements can create short- to medium-term impacts on food security in local communities in mainly two ways (3). First, IDPs can cause a population shock, a sudden increase in population due to forced displacement in a geographical area (4). Such a population shock can also create additional pressure on existing food systems. Hence, the increasing number of IDPs in Yemen has also escalated the pre-existing vulnerabilities, especially in terms of food insecurity. As a country that has always been one of the poorest in the Arab world, Yemen has, for a long time, had to struggle with poverty, unemployment, and inadequate food systems (5). However, the introduction of the conflict made the predicament unbearable by causing obstructed access to food. The plight of people, particularly those who are affected by the conflict, has leveraged these problems, and as a consequence, hunger and malnutrition have become a significant threat to the displaced individuals and host communities (5,6). The hunger problem in Yemen is extremely severe. Based on the United Nations (2022) report, approximately 17 million people, or more than half of Yemen's population, have experienced food insecurity.

In contrast, the majority of them were displaced due to the conflict, and the rest have limited availability of healthy food, thereby becoming more vulnerable to chronic hunger and severe malnutrition (7). The situation is often made worse by migration forced on those displaced people, who not only lose the income they had previously had as farmers and in small businesses but also the ability to become self-reliant. As a consequence, the movement of people (either within Yemen or to the neighboring countries) directly affects the supply of food, jobs, and social benefits, which are essential elements in maintaining the food security status of the people (8).

The dynamics of food insecurity and migration are intricately intertwined and challenging to disentangle. Conflict-driven migration within Yemen and the international movement to the neighboring states have diverse food security challenges (6,9). Migrants not only lose connection to the agricultural sector, animals, and markets but also face income and social barriers when they reach the host areas (10). It has been demonstrated in earlier studies that the problem of food insecurity is more prevalent among conflict migrants than among non-migrants or voluntary migrants, mainly due to the loss of social and economic capital, as well as disruptions to food systems (11). The evidence from other regions affected by conflict, such as Syria, Nigeria, and South Sudan, indicates that forced migration has led to a significant reduction in food security, with displaced people experiencing more intense hunger than the native communities (12,13). Similarly, in Yemen, the displacement caused by the conflict has been a major contributor to the increase in food insecurity (14).

Nonetheless, this critically urgent issue has not even come close to being addressed in the academic realm. The purpose of this study is to investigate the associations between conflict-induced migration and food insecurity in Yemen, using data from the Yemen Multiple Indicator Cluster Survey (MICS) 2022-2023 (15). The paper will help establish an association between migration

as a direct result of a crisis and food insecurity by examining and quantifying two indicators: hunger rates and the frequency of missing meals on a daily basis, particularly in conflict-affected areas. Meanwhile, the study would also be controlled for sociodemographic factors, including gender, education, and urban-rural location, which may play a role in this relationship. The insights of the study would be used to create effective measures for addressing hunger and malnutrition in the refugee population and be an influential factor in the humanitarian response plans in Yemen and other conflict-affected areas across the globe.

Methodology

The study investigates how different types of migration affect food security. This section presents a comprehensive methodology for exploring the relationship of conflict-led migration and food insecurity among Yemeni individuals.

Data Description

The data used in this study are from the Yemen Multiple Indicator Cluster Survey 2022-2023. Yemen's Central Statistical Organization led this comprehensive survey with technical assistance from UNICEF. The survey employed a multi-stage, stratified cluster sampling design. The survey was designed to gather an extensive set of data to assess the living conditions of Yemenis. The survey was conducted at sub-national and rural/urban levels for the twenty-two governorates in Yemen. Due to ongoing conflict and access limitations, minor modifications in some areas were incorporated into the sampling plan. Approximately 20,000 households were targeted for the survey, and the survey questionnaire collected data on migration status and food insecurity. The

migration and food security module were administered to 13,891 individuals. The total number of observations analyzed in this study is 13,891 individuals.

Dependent and Independent Variables

The two principal dependent variables investigated in this study are the incidence of hunger and frequency of not eating for a whole day. The survey questionnaire inquired whether the respondent had been hungry but did not eat due to a lack of money or other resources in the past month and whether the respondent had gone without eating for a whole day because of lack of money or other resources during the same period. These two variables are specified as categorical variables. Both variables are presented as incidences of hunger or not eating for an entire day in the past 30 days (the indicators are count variables). The hunger indicator is coded based on the frequency with which an individual experiences hunger each week: Never (coded as 0), once a week (coded as 1), more than once a week (coded as 2), and almost every week (Coded as 3). For this study, these categories were grouped into No hunger (coded as 0), mild hunger (coded as 1), moderate hunger (coded as 2), and severe hunger (Coded as 3). The Not Eating for a Whole Day indicator was similarly coded. For this study, these categories were coded as follows: Never (coded as 0), Rarely (coded as 1), Sometimes (coded as 2), and Often (Coded as 3).

The focus of this study is how migration due to conflict affects the incidence of food insecurity among Yemeni individuals. The study employs five binary migration variables to examine the impact of migration on food insecurity. The variables are listed below. The MICS survey questionnaire included a module on the respondent's migration status. Two questions are the focus of the study. First, the respondents are asked where they migrated from. The second question was

the reason for migration. Using these questions, 5 variables were created to analyze the effect of migration on food security.

1. Migration due to conflict: This binary variable equal '1' if the individual migrated due to conflict (self-reported, regardless of whether internal or international) and '0' otherwise.
2. Migrating from another country: This binary variable equal '1' if the individual migrated from outside Yemen (as self-reported, regardless of the reason) and '0' otherwise.
3. Migrating from another Governorate: This binary variable equal '1' if the individual migrated from within Yemen (self-reported, regardless of the reason) and ‘0' otherwise.
4. Migrating due to conflict from Another Country: This binary variable equal '1' if the individual migrated due to conflict from another country to Yemen, and '0' otherwise.
5. Migrating due to conflict from another Governorate: This binary variable equal '1' if the individual migrated due to conflict from another Governorate in Yemen and '0' otherwise.

Additional variables are included to control mediating and moderating variables. All the information is given in Table 1. These control variables include:

1. Female: binary variable; if the individual is a female, it's 1, and 0 if male.
2. Age: A categorical variable representing the age of the individual, ranging from under 10 years old to over 60 years old.
3. Household members: A categorical variable representing the number of members in the individual’s household.
4. Education Level: A categorical variable representing the level of education the individual completed.
5. Urban: A binary variable if where the individual lives in an urban area, it’s 1 and 0 if the individual lives in a rural area.

6. Composite wealth index: A standardized continuous variable that represents the wealth level of the individual's household using asset ownership.

These variables were used in the statistical analysis to find the impact of migration, especially migration due to conflict, on the incidence of food insecurity.

Statistical Analysis

The statistical analysis employed two methods to investigate the impact of conflict migration on the incidence of food insecurity. The first part used descriptive statistics analysis, and the second part utilized Negative Binomial Regression analysis.

In the descriptive statistics analysis part, the sample was first illustrated. This displays the part of the sample that was chosen and provides a snapshot of the data used in this study. Secondly, descriptive statistics and Pearson's chi-square tests were used to study the differences in food insecurity among conflict migrants across governorates in Yemen.

The two dependent variables ('Hunger' and 'Not Eating for a Whole Day') are coded as incidences of food insecurity in a month. As the variables are count data, Poisson or Negative Binomial Regression are the best methods for regression analysis. The dependent variables are over-dispersed, indicating that the variance of the indicators exceeds their mean. For this reason, a Negative Binomial Regression method is the most suitable approach in this regard. To investigate if the migration variables with statistical significance explain the model of food insecurity, Akaike Information Criterion, Bayesian Information Criterion, and Likelihood Ratio Test were used. The results of the regression analysis are presented as the Incidence Rate Ratio (adjusted for differences in governorates), along with the corresponding p-value and 95% confidence interval.

Results

Table 1 presents a snapshot of the sample's characteristics used for analysis. In the study, the distribution was almost equal between male (46.86%) and female (53.14%) respondents. There was a slightly greater number of respondents living in urban areas (58.68%) than in rural areas (41.32%). The highest sample of respondents resided in Sana's City (19.51%), Aden (9.13%), Sa'ada (8.89%), Ibb (6.57%), and Abyan (6.12%) governorates. About 54% of the respondents were aged between 11 and 13 years old. About 61% of the respondents had at least a Lower Secondary Level of Education. The highest proportion of the sample was in the wealthiest quintile (40.04%) of households. Approximately, 24% and 12% of respondents experienced hunger or did not eat for a whole day, respectively.

Table 1: Descriptive Statistics

	N	***%***
**Sex of Respondent**		
Male	6,509	46.86
Female	7,382	53.14
**Area**		
Urban	8,151	58.68
Rural	5,740	41.32
**Governorate**		
Ibb	1,377	6.57
Abyan	1,282	6.12
Sana'a City	4,087	19.51
Al Bayda	838	4.00
Taizz	1,094	5.22
Al Jawf	302	1.44
Hajjah	490	2.34
Al Hudaydah	536	2.56
Hadramaut	556	2.65
Dhamar	621	2.97
Shabwah	735	3.51
Sa'ada	1,861	8.89
Sana'a	540	2.58
Aden	1,913	9.13

Lahj	345	1.65
Marib	688	3.28
Al Mahwit	725	3.46
Al Maharah	312	1.49
Amran	989	4.72
Al Dhale'e	478	2.28
Raymah	955	4.56
Socotra	220	1.05
**Age**		
0-10 Years	1325	9.54%
11 - 20 Years	3760	27.07%
21 - 30 Years	3803	27.38%
31-40 Years	2658	19.13%
41-50 Years	1436	10.34%
51-60 Years	606	4.36%
61 and Above Years	303	2.18%
**Education Level**		
Pre-primary or none	45	0.32
Primary Education	5,414	38.97
Lower Secondary Education	2,878	20.72
Upper Secondary Education	3,647	26.25
Higher	1,891	13.61
**Wealth Quintile**		
Poorest	1,151	8.29
Second	1,204	8.67
Middle	1,959	14.1
Fourth	4,015	28.9
Richest	5,562	40.04
**Hunger (In the past month)**		
No Hunger	10,536	75.85
Mild Hunger	1,293	9.31
Moderate Hunger	1,135	8.17
Severe Hunger	927	6.67
**Without Eating for a whole day (In the past month)**		
Never	12,243	88.14
Rarely	784	5.64
Sometimes	536	3.86
Often	328	2.36

Table 2 represents the level of hunger across the governorates. It shows that the highest levels of severe hunger among conflict migrants were in Al Mahwit (44.94%) and Dhamar (29.03%). The highest levels of mild hunger were in Al Jawf (47.62%), Al Hudaydah (34.44%), and Al Maharah

governorates. The highest levels of no hunger among conflict migrants were from the Ibb (77.27%), Abyan (73.36%), and Taizz (68.24%) governorates.

Table 2: Level of Hunger across governorates that migrated due to conflict

	**No Hunger**	**Mild Hunger**	**Moderate Hunger**	**Severe Hunger**
Al Mahwit	39.33%	8.99%	6.74%	44.94%
Dhamar	46.77%	11.29%	12.90%	29.03%
Al Jawf	0.00%	47.62%	23.81%	28.57%
Al Bayda	51.69%	11.02%	9.32%	27.97%
Marib	50.46%	25.23%	4.62%	19.69%
Al Dhale'e	58.82%	13.73%	7.84%	19.61%
Ibb	77.27%	1.82%	3.64%	17.27%
Lahj	63.41%	7.32%	12.20%	17.07%
Aden	53.02%	8.05%	22.15%	16.78%
Amran	38.53%	14.68%	32.11%	14.68%
Sana'a	61.22%	4.08%	20.41%	14.29%
Hajjah	61.17%	6.80%	22.33%	9.71%
Raymah	57.41%	18.52%	15.74%	8.33%
Taizz	68.24%	16.47%	7.65%	7.65%
Al Hudaydah	38.89%	34.44%	21.11%	5.56%
Abyan	73.36%	9.17%	12.01%	5.46%
Al Maharah	57.14%	30.61%	8.16%	4.08%
Sana'a City	91.24%	1.38%	4.61%	2.76%
Shabwah	91.18%	0.00%	7.35%	1.47%
Sa'ada	80.16%	14.49%	4.25%	1.10%
Hadramaut	91.30%	0.00%	8.70%	0.00%
Socotra	100.00%	0.00%	0.00%	0.00%
Pearson Chi2 p-value	< 0.001			

Table 3 represents the frequency of going without eating for a whole day across governorates that migrated due to conflict. Compared to hunger, the incidence of not eating for an entire day is lower. Conflict migrants in Lahj (19.51%) and Al Jawf (14.29%) had the highest proportion of often going without eating for a whole day. The highest levels of rarely going without eating for an entire day were among conflict migrants in Al Jawf (47.62%), Al Maharah (30.61%), and Al Dhale'e (27.45%).

Table 3: Frequency of going without eating for a whole day across governorates who migrated due to conflict

	**Never**	**Rarely**	**Sometimes**	**Often**
Lahj	68.29%	4.88%	7.32%	19.51%
Al Jawf	0.00%	47.62%	38.10%	14.29%
Amran	53.21%	24.77%	9.17%	12.84%
Marib	70.15%	14.46%	3.08%	12.31%
Aden	79.87%	1.34%	7.38%	11.41%
Sana'a	73.47%	2.04%	14.29%	10.20%
Taizz	81.76%	7.65%	2.35%	8.24%
Abyan	84.50%	2.18%	8.95%	4.37%
Al Bayda	81.36%	3.39%	11.02%	4.24%
Ibb	94.55%	0.00%	1.82%	3.64%
Al Hudaydah	82.22%	12.22%	2.22%	3.33%
Dhamar	75.81%	19.35%	3.23%	1.61%
Shabwah	94.12%	0.00%	4.41%	1.47%
Sa'ada	88.03%	9.45%	1.42%	1.10%
Al Mahwit	59.55%	12.36%	28.09%	0.00%
Raymah	73.15%	12.04%	14.81%	0.00%
Hajjah	73.79%	11.65%	14.56%	0.00%
Al Dhale'e	58.82%	27.45%	13.73%	0.00%
Hadramaut	91.30%	0.00%	8.70%	0.00%
Al Maharah	61.22%	30.61%	8.16%	0.00%
Sana'a City	97.70%	0.00%	2.30%	0.00%
Socotra	100.00%	0.00%	0.00%	0.00%
Pearson Chi2 p-value	< 0.001			

Table 4 presents the results of the negative binomial regression, illustrating the impact of experiencing hunger or going without food for a whole day on Yemeni individuals. Individuals who migrated due to conflict significantly increased the incidence of hunger (IRR: 1.262, 95% CI: 1.111–1.435) and reported not eating for the whole day (IRR: 1.251, 95% CI: 1.103–1.516). However, individuals migrating to Yemen had a lower incidence of food insecurity (Hunger: IRR: 0.638, 95% CI: 0.480 – 0.848; Without Eating: IRR: 0.600, 95% CI: 0.367–0.981). Migrating from another governorate did not have a statistically significant impact on the incidence of food insecurity. If the individual migrated due to conflict and from another country, with statistical robustness, it increases the risk of incidence of hunger (IRR: 2.100, 95% CI: 1.279–3.450) by approximately more than two-folds and the likelihood of going without eating (IRR: 6.001, 95%

CI: 2.951–12.206) by about six-folds. Migrating due to conflict from another governorate did not have a statistically significant effect on the incidence of hunger among individuals. Migrating due to conflict from another governorate had a statistically significant impact on the incidence of an individual going without eating for a whole day (IRR: 1.312, p-value < 0.050).

Among the confounding variables, being female is associated with a lower incidence of going hungry (IRR: 0.871, 95% CI: 0.812–0.935) or not eating for a whole day (IRR: 0.782, 95% CI: 0.700–0.873). The education level had a statistically significant effect on decreasing the incidence of hunger (IRR: 0.897, 95% CI: 0.867-0.929) and going without eating for a whole day (IRR: 0.867, 95% CI: 0.821–0.916). Similarly, if the individual is from a wealthier household, there is a significant impact on decreasing the incidence of hunger (IRR: 0.526, 95% CI: 0.491–0.564) and going without eating for a whole day (IRR: 0.436, 95% CI: 0.396–0.481). Individuals living in urban areas are about 2.231- and 3.657-times higher incidence to go hungry (IRR: 2.231, 95% CI: 1.990–2.501) or go without eating for a whole day (IRR: 3.657, 95% CI: 3.086–4.335), respectively.

Table 4: The result of the Negative Binomial Regression

	Hunger			Without Eating		
	IRR	95% Confidence Interval	P-value	IRR	95% Confidence Interval	P-value
Migration due to conflict	1.262	(1.111, 1.435)	< 0.001	1.251	(1.032, 1.516)	< 0.050
Migrating from another country	0.638	(.480, .848)	<0.010	0.600	(.367, .981)	< 0.050
Migrating from another Governorate	1.003	(.914, 1.099)	NS	0.889	(.761, 1.039)	NS
Migrating due to conflict from another Country	2.100	(1.279, 3.450)	<0.001	6.001	(2.951, 12.206)	< 0.001
Migrating due to conflict from another Governorate	1.014	(.864, 1.188)	NS	1.312	(1.032, 1.667)	< 0.050
**Control variables**						
Female	0.871	(.812, .935)	< 0.001	0.782	(.700, .873)	< 0.001

Age	0.998	(.996, 1.001)	NS	0.995	(.990, .999)	< 0.010
Household Members	1.002	(.992, 1.012)	NS	0.996	(.981, 1.011)	NS
Education Level	0.897	(.867, .929)	< 0.001	0.867	(.821, .916)	< 0.001
Urban	2.231	(1.990, 2.501)	< 0.001	3.657	(3.086, 4.335)	< 0.001
Composite Wealth Index	0.526	(.491, .564)	< 0.001	0.436	(.396, .481)	< 0.001

IRR: Incidence Rate Ratio (Adjusted for Governorate differences)

Discussion

This research examined how war and conflict-induced migration affected food security in Yemen, utilizing data from the Yemen Multiple Indicator Cluster Survey (MICS) 2022-2023. The investigation unveiled that migration, especially for conflict-related reasons, had a considerable impact on both the prevalence of hunger and the frequency of going without food for an entire day. The results align with the findings of the current literature, which primarily emphasize the detrimental effects of forced migration on food security. It is well-documented that the exodus of populations from their homes and their relocation significantly increases their likelihood of experiencing food insecurity, as they are deprived of access to the means of earning their living, food, and other social services (11).

The findings revealed that people displaced due to conflict experienced a significantly higher likelihood of hunger and skipping meals throughout the day. This thus provides support to the hypothesis that conflict-led displacement reduces food accessibility, deepens economic instability, and intensifies the need for humanitarian aid, which is often insufficient in the majority of the cases (12). The results align with research from other conflict-affected areas, such as Syria and South Sudan, where involuntary migration has been found to contribute to higher food insecurity

due to the collapse of local economies, mass displacement from agricultural land, and the loss of income-generating activities (13).

The data also indicate that the situation of people moving from one country to another due to war or conflict is the most serious problem in food security, with a significantly higher rate of starvation and people not having access to food for a day. This result is consistent with previous studies that showed that international migrants are usually plagued with extra social and economic woes like legal obstacles, discrimination, and exclusion from the social safety net, which underpin their food and nutrition insecurity (8). These issues may also emerge as a result of the struggles migrants face when integrating into communities that are not easily accessible to the host communities, leading to an aggravation of their food insecurity (11).

However, it was found that migration from another part of the governorate within Yemen did not have a statistically significant effect on the incidence of lack of food or going without food for the entire day, which suggests that local displacement within a country may not have an impact as severe as international migration on the food security of a nation. On the contrary, migration due to conflict from a different governorate was statistically significant for the incidence of going without a meal for the whole day, thus showing that even internal displacement can affect access to food, albeit with less impact compared to international migration. These results are consistent with previous literature on food insecurity and migration. Migration driven by conflicts often results in the loss of access to food, which alters social and economic structures and places additional burdens on host communities (11). There is also evidence that these incidences of food insecurity are exacerbated in vulnerable regions, such as Africa, the Middle East, and Eastern Europe (11). Research on forced migrants in Nigeria and Jordan shows that migrants lose their social, cultural, human, and economic capital, which is essential to maintaining food security. It

has also been demonstrated that the influx of migrants into host communities strains both cultural and economic capital within these communities (12,13).

Among the determinants, a few variables were observed to be confounding factors of food insecurity as well. It was noticeable that the female participants were statistically less prone to going to sleep hungry or starving for a whole day. This suggests that women may be the primary figures in eliminating food insecurity in families, as they are typically responsible for household food management (16–18). This result is similar to many other studies that have recognized women as the main authority in handling the food security of their households, especially in areas with constrained resources (19).

The influence of education on food security was also observed, as respondents with higher levels of education had a lower risk of hunger and food insecurity. This finding aligns with earlier research that has emphasized the role of education in promoting economic stability, access to resources, and nutritional knowledge, all of which are key factors in preventing food insecurity (20–23). Educational attainment reduces food insecurity by equipping individuals with literacy skills, nutrition education, and increasing their likelihood of financial stability (19). On the same note, a statistically significant decrease in the ratio of food insecurity was observed in individuals with higher incomes, thus implying the widely observed connection between income and food security (12).

Living in the urban areas led to a higher incidence of hunger and going without food as urban migrants face numerous hurdles in these cities, where the scarcity of resources is severe, and the number of self-sufficiency opportunities in food production is low (24,25). A multitude of studies have indicated that employment issues are the primary cause of stress for urban migrants, and due to the escalating living costs associated with the job, they struggle more to access sufficient food

(26–28). The rural-urban division of food insecurity highlights the importance of understanding the role of the social environment in the interrelationship between migration and food insecurity. Previous literature has portrayed urban sprawl and inefficient city structures in sub-Saharan Africa as leading to socioeconomic segregation, which affects food accessibility for migrants (29).

Finally, the research explored that the wealth index not only had a significant protective effect on food security but also those citizens who were better off in terms of social class on average were the ones who encountered fewer physical sensations of hunger and no experiences of missing a meal. This highlights the importance of financial well-being in achieving food security, especially in conflict-affected areas where access to food is often highly constrained (30).

The research has highlighted that displacements caused by war and conflict are the primary cause of the increased food insecurity in Yemen. The findings of this study have significant policy and humanitarian implications, indicating the necessity of direct aid for conflict migrants and those migrating from abroad, in particular, and suggest addressing the deeper socioeconomic issues that lead to food insecurity in Yemen.

Conclusion and Policy Recommendation

This research presents the insight that the upsurge in hunger among conflict-affected people in Yemen causes significant food crises, especially for displaced individuals who experience greater hunger and are more likely to forgo food for an entire day. The movement of people due to war and conflict presents a challenge not only for the internal inhabitants but also for those crossing borders, which in turn seems to influence the severity of the problem in the latter case. The findings of this study also indicate that factors such as gender, education, wealth, and the urban-rural

location of the population are highly significant in determining food security. According to the findings of this study, women, individuals with higher education, and the affluent were not the minority of people who faced food scarcity; however, urban dwellers showed an increasing vulnerability. This evidence is consistent with the existing literature, which indicates that conflicts can hinder food access, leading to economic instability and necessitating more humanitarian assistance. This research highlights the complex issue of food insecurity in conflict-prone territories, underscoring that migration, particularly forced migration, is a significant contributor to people's exposure to risk of food insecurity. In Yemen, food access interruption due to displacement is accompanied by social and economic discrepancies that, beyond that, nothing else makes even more the difficulties the displaced are facing. It is for these reasons that addressing the issue of hunger among migrants should be a comprehensive process to provide both prompt relief and sustainable solutions simultaneously. Future research should investigate the pathways through which conflict-induced migration affects food insecurity.

Several policy recommendations flow from this research to mitigate the food insecurity faced by the conflict migrants in Yemen. The first and most pressing issue is that humanitarian programs should be boosted, with a focus on the migrant population, especially foreign migrants. These groups face numerous unprecedented challenges that hinder their access to food, stemming from legal, social, and economic inequalities. Thus, the aid program should not only deliver essential food items in an emergency but also explore long-term food security strategies, such as providing nutritional treatments and establishing a sustainable food distribution system in the region.

Second, it is necessary to focus on creating sustainable jobs for the people forced to leave, which is especially important in cities where migrants are at the highest risk. Enabling migrants to be part of the local economy through job skills training, job market participation, and encouragement of

small enterprises can result in better income levels for them and more stable food security. The problem of excessive competition for resources and high living expenses in urban areas should be given special attention owing to the consequences of urban migration. The development of a good transportation system with cities from the periphery might reduce the rate of urban migration in Yemen.

Thirdly, enhancing the availability of education, particularly for women and children, has paramount importance for sustainable food security. Knowledge gained from education enables people to secure decent and stable incomes and access to the better nutrition and food security. Hence, educational interventions should incorporate elements of both formal education and training in nutrition, agricultural practices, and food security, thereby enabling displaced individuals to possess the necessary information for informed decision-making regarding provision of food. Moreover, assimilating gender-sensitive strategies into food security and migration policies is crucial. Women, children, and old age people have always been the major sufferers within families; therefore, if women are empowered through the provision of resources, education, and economic opportunities, they can substantially contribute to food security within their families, resulting in empowerment through access to land, credit, and entrepreneurial training. This will help them manage food more effectively and assist their families in coping with everyday challenges.

In conclusion, this study reiterates the necessity for comprehensive approaches that address the pressing issue of food security for displaced people in Yemen in a sustainable manner. Humanitarian aid, job provision, education, and gender activities are to be combined into a comprehensive strategy for food security intervention. The realization of these recommendations can positively influence the resilience of the affected communities, the reduction of food

insecurity, and the recovery of Yemen and similar conflict-affected regions in the long run by the governments and humanitarian organizations

Data Availability Statement: Data will be made available on request.

References:

1. Watol BS. The Post-2011 Yemen Internationalized Civil War: It's National, Regional and Global Implications. European Journal of Arts, Humanities and Social Sciences. 2024 Jul 1;1(4):3–25.

2. Internal Displacement Monitoring Centre. Global Report on Internal Displacement 2024 [Internet]. Internal Displacement Monitoring Centre; 2024 [cited 2025 May 11]. Available from: https://www.internal-displacement.org/global-report/grid2024

3. Verme P, Schuettler K. The Impact of Forced Displacement on Host Communities. World Bank Policy Research Working Paper (8727) [Internet]. 2019 [cited 2025 May 15]; Available from: https://documents1.worldbank.org/curated/en/654811549389186755/pdf/WPS8727.pdf

4. Becker SO, Ferrara A. Consequences of forced migration: A survey of recent findings. Labour Economics. 2019 Aug;59:1–16.

5. Tandon S, Vishwanath T. The evolution of poor food access over the course of the Conflict in Yemen. World Development. 2020 Jun;130:104922.

6. Sihaloho RAH, Prabowo TB, Kusuma RP. Food Crisis in Yemen: The Roles of Food and Agriculture Organization (FAO) from 2015 to 2020. NSJIS. 2022 Jul 23;5(1):59–74.

7. United Nations. UN Yemen Country Results Report 2022. 2022.

8. Al-Waleedi AA, Bin-Ghouth AS. Malnutrition among hospitalized children 12–59 months of age in Abyan and Lahj Governorates / Yemen. BMC Nutr. 2022 Aug 12;8(1):78.

9. Demurtas A, and Occansey Agbeko A. A comparative analysis of two food security and nutrition policies in Yemen between 2018 and 2023: agricultural livelihoods and multisectoral nutrition plan. Global Security: Health, Science and Policy. 2025 Dec 31;10(1):2499453.

10. Dureab F, Al-Falahi E, Ismail O, Al-Marhali L, Al Jawaldeh A, Nuri NN, et al. An Overview on Acute Malnutrition and Food Insecurity among Children during the Conflict in Yemen. Children. 2019 Jun;6(6):77.

11. Iriti M, Vitalini S. Climate change, natural disasters, armed conflicts and migrations at the crossroads between food and nutrition insecurity and undernourishment. FFS. 2025 Jan 2;5(1):1–5.

12. Bertassello L, Winters P, Müller MF. Access to global wheat reserves determines country-level vulnerability to conflict-induced Ukrainian wheat supply disruption. Nat Food. 2023 Aug 3;4(8):673–6.

13. Hassan Onimisi Abu, Megan K Blake. Characterization of food insecurity among the forced migrants in northern Nigeria. Magna Sci Adv Res Rev. 2024 Jan 30;10(1):100–14.

14. Kandeh J, Kumar L. Developing a Relative Ranking of Social Vulnerability of Governorates of Yemen to Humanitarian Crisis. IJGI. 2015 Sep 29;4(4):1913–35.

15. Central Statistical Organization (CSO), United Nations Children's Fund (UNICEF). Yemen multiple Indicator Cluster Survey 2022–2023, Survey findings Report. emen: Central Statistical Organization and United Nations Children's Fund; 2023.

16. Hernández-Moreno A, Gutiérrez-Gutiérrez F, Celedón-Celis N, Girona-Gamarra M, Hochstetter-Diez J. Analysis of Public Policies on Food Security for Older Mapuche Adults in Rural Areas. Foods. 2025 Mar 19;14(6):1055.

17. Hernandez DC. The impact of cumulative family risks on various levels of food insecurity. Social Science Research. 2015 Mar;50:292–302.

18. Hernandez DC, Kim BR, Brooks FP, Gundersen C. The association between hunger-coping economic tradeoffs and food insecurity among female recipients of charitable food assistance. Appetite. 2025 Jan;204:107733.

19. Fitawek W, Hendriks SL. Assessing the impact of inequality on food security. In: Hendriks SL, Babu SC, editors. Handbook on Public Policy and Food Security [Internet]. Edward Elgar Publishing; 2024 [cited 2025 May 15]. p. 260–7. Available from: https://www.elgaronline.com/view/book/9781839105449/book-part-9781839105449-30.xml

20. Tauseef S. The Importance of Nutrition Education in Achieving Food Security and Adequate Nutrition of the Poor: Experimental Evidence from Bangladesh*. Oxf Bull Econ Stat. 2022 Feb;84(1):241–71.

21. Burchi F. Child nutrition in Mozambique in 2003: The role of mother's schooling and nutrition knowledge. Economics & Human Biology. 2010 Dec;8(3):331–45.

22. Burchi F, De Muro P. From food availability to nutritional capabilities: Advancing food security analysis. Food Policy. 2016 Apr;60:10–9.

23. Islam MdS. Do education and health influence economic growth and food security Evidence from Bangladesh. IJHD. 2020;6(1):59.

24. Sohel MdS, Hossen MdS, Hossain B, Sifullah MdK, Galib A. Mapping Out the Food Insecurity of Urban Floating Labour in Mega City of Dhaka: a Qualitative Study. Int Journal of Com WB. 2024 Jun 1;7(2):213–35.

25. Hoque F, Khan MdA, Urme IA, Sultana R, Al Imran S. Food Security Status and Associated Drivers Among Climate Migrant Households in Bangladesh: Insight From Urban Informal Settlements. Food and Energy Security. 2024;13(6):e70020.

26. Wirtz AL, Stevenson M, Guillén JR, Ortiz J, Barriga Talero MÁ, Page KR, et al. Persistent Food Insecurity and Material Hardships: A Latent Class Analysis of Experiences among Venezuelan Refugees and Migrants in Urban Colombia. Nutrients. 2024 Jan;16(7):1060.

27. Borku AW, Utallo AU, Tora TT. The level of food insecurity among urban households in southern Ethiopia: A multi-index-based assessment. Journal of Agriculture and Food Research. 2024 Mar 1;15:101019.

28. Crush J, Frayne ,Bruce, and Pendleton W. The Crisis of Food Insecurity in African Cities. Journal of Hunger & Environmental Nutrition. 2012 Apr 1;7(2–3):271–92.

29. Hemerijckx LM, Janusz K, Van Emelen S, Tumwesigye S, Davis J, Lwasa S, et al. Food accessibility of different socioeconomic groups in sub-Saharan African cities: a mixed-method analysis in Kampala, Uganda. Food Sec. 2022 Jun;14(3):677–94.

30. Arshad A. Impact of financial inclusion on food security: evidence from developing countries. IJSE. 2022 Feb 21;49(3):336–55.

Click here to access/download
Supporting Information
Supporting Information (1).docx

From Displacement to Hunger: How Migration Due to Conflict Affects Food Security in Yemen

Sayed Jubair Bin Hossain[1]¶

Niaz Makhdum[2]¶

Maruf Hasan Rumi[3]*¶

[1] Department of Economics, University of Dhaka, Dhaka, Bangladesh.
Email: sjb.hossain@du.ac.bd; Orcid: https://orcid.org/0009-0001-3599-5293
[2] Department of Public Administration, Begum Rokeya University, Rangpur, Bangladesh.
Email: niaz.makhdum1995@gmail.com ; Orcid: https://orcid.org/0000-0003-0885-605X
[3] Department of Public Administration, University of Dhaka, Dhaka, Bangladesh.
Email: marufhasanrumi@du.ac.bd; Orcid: https://orcid.org/0000-0003-1752-5615

***Corresponding author**
Email: marufhasanrumi@du.ac.bd

From Displacement to Hunger: How Migration Due to Conflict Affects Food Security in Yemen

Abstract

Background: Conflict-induced migration is a key driver of global food insecurity. Over the past decade, prolonged conflicts have displaced millions of people. This crisis plunged millions into acute hunger, deepening existing vulnerabilities and severely restricting access to food and livelihoods across Yemen.

Objective: This study aims to investigate the relationship between conflict-induced migration and food insecurity in Yemen, focusing on how displacement exacerbates hunger and malnutrition among affected populations.

Methodology: This study analyzed responses from 13,891 individuals across 22 governorates, using data from the Yemen Multiple Indicator Cluster Survey (MICS) 2022–2023. The two primary indicators of food insecurity — specifically, hunger frequency and instances of not eating for a full day — were examined. Additionally, Negative Binomial Regression was employed to model the over-dispersed count data while controlling for factors such as gender, age, education, household size, urban-rural location, and wealth.

Results: The findings revealed that approximately 24% of respondents experienced hunger, while about 12% went without food for an entire day. Moreover, conflict-induced migration substantially augmented the incidence of hunger (IRR = 1.262; 95% CI: 1.111–1.435) and going without eating for an entire day (IRR= 1.251; 95% CI: 1.032-1.516), though international migration to Yemen decreased food insecurity. Additionally, being female, having higher education, and belonging to wealthier households were protective factors against food insecurity, while urban residence

significantly increased the likelihood of hunger (IRR = 2.231; 95% CI: 1.990- 2.501) and meal skipping (IRR = 3.657; 95% CI: 3.086-4.335).

Conclusion: Conflict-induced displacement is associated with worsened food insecurity, escalating hunger, and deprivation among displaced people, underscoring the immediate necessity of targeted humanitarian interventions in Yemen. Policy measures must prioritize the re-establishment of food systems and livelihoods in affected regions to alleviate the intensifying hunger crisis.

Keywords: *Displacement, Hunger, Migration, Conflict, Food Security, Yemen.*

Introduction

Migration caused by conflict has become a leading global crisis of the 21st century, with severe socioeconomic and humanitarian effects. The armed conflict in Yemen, ongoing since 2014, has resulted in the displacement of millions of people, both internally and externally (1). The total number of people living in internal displacement increased by 51% over the past five years, reaching a record high of 75.9 million people across 116 countries at the end of 2023. Of the 75.9 million Internally Displaced People (IDPs) at the end of 2023 globally, 68.3 million were displaced by conflict and violence, and in Yemen, this number is 4.5 million (2). Forced displacements can create short- to medium-term impacts on food security in local communities in mainly two ways (3). First, IDPs can cause a population shock, a sudden increase in population due to forced displacement in a geographical area (4). Such a population shock can also create additional pressure on existing food systems. Hence, the increasing number of IDPs in Yemen has also escalated the pre-existing vulnerabilities, especially in terms of food insecurity. As a country that has always been one of the poorest in the Arab world, Yemen has, for a long time, had to struggle with poverty, unemployment, and inadequate food systems (5). However, the introduction of the conflict made the predicament unbearable by causing obstructed access to food. The plight of people, particularly those who are affected by the conflict, has leveraged these problems, and as a consequence, hunger and malnutrition have become a significant threat to the displaced individuals and host communities (5,6). The hunger problem in Yemen is extremely severe. Based on the United Nations (2022) report, approximately 17 million people, or more than half of Yemen's population, have experienced food insecurity.

In contrast, the majority of them were displaced due to the conflict, and the rest have limited availability of healthy food, thereby becoming more vulnerable to chronic hunger and severe malnutrition (7). The situation is often made worse by migration forced on those displaced people, who not only lose the income they had previously had as farmers and in small businesses but also the ability to become self-reliant. As a consequence, the movement of people (either within Yemen or to the neighboring countries) directly affects the supply of food, jobs, and social benefits, which are essential elements in maintaining the food security status of the people (8).

The dynamics of food insecurity and migration are intricately intertwined and challenging to disentangle. Conflict-driven migration within Yemen, and the international movement to the neighboring states have diverse food security challenges (6,9). Migrants not only lose connection to the agricultural sector, animals, and markets, but also face income and social barriers when they reach the host areas (10). It has been demonstrated in earlier studies that the problem of food insecurity is more prevalent among conflict migrants than among non-migrants or voluntary migrants, mainly due to the loss of social and economic capital, as well as disruptions to food systems (11). The evidence from other regions affected by conflict, such as Syria, Nigeria, and South Sudan, indicates that forced migration has led to a significant reduction in food security, with displaced people experiencing more intense hunger than the native communities (12,13). Similarly, in Yemen, the displacement caused by the conflict has been a major contributor to the increase in food insecurity (14).

Nonetheless, this critically urgent issue has not even come close to being addressed in the academic realm. The purpose of this study is to investigate the associations between conflict-induced migration and food insecurity in Yemen, using data from the Yemen Multiple Indicator Cluster Survey (MICS) 2022-2023 (15). The paper will help establish an association between migration

as a direct result of a crisis and food insecurity by examining and quantifying two indicators: hunger rates and the frequency of missing meals on a daily basis, particularly in conflict-affected areas. Meanwhile, the study would also be controlled for sociodemographic factors, including gender, education, and urban-rural location, which may play a role in this relationship. The insights of the study would be used to create effective measures for addressing hunger and malnutrition in the refugee population, and be an influential factor in the humanitarian response plans in Yemen and other conflict-affected areas across the globe.

Methodology

The study investigates how different types of migration affect food security. This section presents a comprehensive methodology for exploring the relationship of conflict-led migration and food insecurity among Yemeni individuals.

Data Description

The data used in this study are from the Yemen Multiple Indicator Cluster Survey 2022-2023. Yemen's Central Statistical Organization led this comprehensive survey with technical assistance from UNICEF. The survey employed a multi-stage, stratified cluster sampling design. The survey was designed to gather an extensive set of data to assess the living conditions of Yemenis. The survey was conducted at sub-national and rural/urban levels for the twenty-two governorates in Yemen. Due to ongoing conflict and access limitations, minor modifications in some areas were incorporated into the sampling plan. Approximately 20,000 households were targeted for the survey, and the survey questionnaire collected data on migration status and food insecurity. The

migration and food security module were administered to 13,891 individuals. The total number of observations analyzed in this study is 13,891 individuals.

Dependent and Independent Variables

The two principal dependent variables investigated in this study are the incidence of hunger and frequency of not eating for a whole day. The survey questionnaire inquired whether the respondent had been hungry but did not eat due to a lack of money or other resources in the past month~~,~~ and ~~if~~whether the respondent had gone without eating for a whole day because of lack of money or other resources during the same period. These two variables are specified as categorical variables. Both variables are presented as incidences of hunger or not eating for an entire day in the past 30 days (the indicators are count variables). The hunger indicator is coded based on the frequency with which an individual experiences hunger each week: Never (coded as 0), once a week (coded as 1), more than once a week (coded as 2), and almost every week (Coded as 3). For this study, these categories were grouped into No hunger (coded as 0), mild hunger (coded as 1), moderate hunger (coded as 2), and severe hunger (Coded as 3). The Not Eating for a Whole Day indicator was similarly coded. For this study, these categories were coded as follows: Never (coded as 0), Rarely (coded as 1), Sometimes (coded as 2), and Often (Coded as 3).

The focus of this study is how migration due to conflict affects the incidence of food insecurity among Yemeni individuals. The study employs five binary migration variables to examine the impact of migration on food insecurity. The variables are listed below. The MICS survey questionnaire included a module on the respondent's migration status. Two questions are the focus of the study. First, the respondents are asked where they migrated from. The second question was

the reason for migration. Using these questions, 5 variables were created to analyze the effect of migration on food security.

1. Migration due to conflict: This binary variable equal '1' if the individual migrated due to conflict (self-reported, regardless of whether internal or international) and '0' otherwise.
2. Migrating from another country: This binary variable equal '1' if the individual migrated from outside Yemen (as self-reported, regardless of the reason) and '0' otherwise.
3. Migrating from another Governorate: This binary variable equal '1' if the individual migrated from within Yemen (self-reported, regardless of the reason) and '0' otherwise.
4. Migrating due to conflict from Another Country: This binary variable equal '1' if the individual migrated due to conflict from another country to Yemen, and '0' otherwise.
5. Migrating due to conflict from another Governorate: This binary variable equal '1' if the individual migrated due to conflict from another Governorate in Yemen and '0' otherwise.

Additional variables are included to control mediating and moderating variables. All the information is given in Table 1. These control variables include:

1. Female: binary variable; if the individual is a female, it's 1, and 0 if male.
2. Age: A categorical variable representing the age of the individual, ranging from under 10 years old to over 60 years old.
3. Household members: A categorical variable representing the number of members in the individual's household.
4. Education Level: A categorical variable representing the level of education the individual completed.
5. Urban: A binary variable if where the individual lives in an urban area, it's 1 and 0 if the individual lives in a rural area.

6. Composite wealth index: A standardized continuous variable that represents the wealth level of the individual’s household using asset ownership.

These variables were used in the statistical analysis to find the impact of migration, especially migration due to conflict, on the incidence of food insecurity.

Statistical Analysis

The statistical analysis employed two methods to investigate the impact of conflict migration on the incidence of food insecurity. The first part used descriptive statistics analysis, and the second part utilized Negative Binomial Regression analysis.

In the descriptive statistics analysis part, the sample was first illustrated. This displays the part of the sample that was chosen and provides a snapshot of the data used in this study. Secondly, descriptive statistics and Pearson's chi-square tests were used to study the differences in food insecurity among conflict migrants across governorates in Yemen.

The two dependent variables (‘Hunger’ –and ‘Not Eating for a Whole Day’) are coded as incidences of food insecurity in a month. As the variables are count data, Poisson or Negative Binomial Regression are the best methods for regression analysis. The dependent variables are over-dispersed, indicating that the variance of the indicators exceeds their mean. For this reason, a Negative Binomial Regression method is the most suitable approach in this regard. To investigate if the migration variables with statistical significance explain the model of food insecurity, Akaike Information Criterion, Bayesian Information Criterion, and Likelihood Ratio Test were used. The results of the regression analysis are presented as the Incidence Rate Ratio (adjusted for differences in governorates), along with the corresponding p-value and 95% confidence interval.

Results

Table 1 presents a snapshot of the sample's characteristics used for analysis. In the study, the distribution was almost equal between male (46.86%) and female (53.14%) respondents. There was a slightly greater number of respondents living in urban areas (58.68%) than in rural areas (41.32%). The highest sample of respondents resided in Sana's City (19.51%), Aden (9.13%), Sa'ada (8.89%), Ibb (6.57%), and Abyan (6.12%) governorates. About 54% of the respondents were aged between 11 and 13 years old. About 61% of the respondents had at least a Lower Secondary Level of Education. The highest proportion of the sample was in the wealthiest quintile (40.04%) of households. Approximately, 24% and 12% of respondents experienced hunger or did not eat for a whole day, respectively.

Table 1: Descriptive Statistics

	N	***%***
**Sex of Respondent**		
Male	6,509	46.86
Female	7,382	53.14
**Area**		
Urban	8,151	58.68
Rural	5,740	41.32
**Governorate**		
Ibb	1,377	6.57
Abyan	1,282	6.12
Sana'a City	4,087	19.51
Al Bayda	838	4.00
Taizz	1,094	5.22
Al Jawf	302	1.44
Hajjah	490	2.34
Al Hudaydah	536	2.56
Hadramaut	556	2.65
Dhamar	621	2.97
Shabwah	735	3.51
Sa'ada	1,861	8.89
Sana'a	540	2.58
Aden	1,913	9.13

Formatted Table

Lahj	345	1.65
Marib	688	3.28
Al Mahwit	725	3.46
Al Maharah	312	1.49
Amran	989	4.72
Al Dhale'e	478	2.28
Raymah	955	4.56
Socotra	220	1.05
**Age**		
0-10 Years	1325	9.54%
11 - 20 Years	3760	27.07%
21 - 30 Years	3803	27.38%
31-40 Years	2658	19.13%
41-50 Years	1436	10.34%
51-60 Years	606	4.36%
61 and Above Years	303	2.18%
**Education Level**		
Pre-primary or none	45	0.32
Primary Education	5,414	38.97
Lower Secondary Education	2,878	20.72
Upper Secondary Education	3,647	26.25
Higher	1,891	13.61
**Wealth Quintile**		
Poorest	1,151	8.29
Second	1,204	8.67
Middle	1,959	14.1
Fourth	4,015	28.9
Richest	5,562	40.04
**Hunger (In the past month)**		
No Hunger	10,536	75.85
Mild Hunger	1,293	9.31
Moderate Hunger	1,135	8.17
Severe Hunger	927	6.67
**Without Eating for a whole day (In the past month)**		
Never	12,243	88.14
Rarely	784	5.64
Sometimes	536	3.86
Often	328	2.36

Table 2 represents the level of hunger across the governorates. It shows that the highest levels of severe hunger among conflict migrants were in Al Mahwit (44.94%) and Dhamar (29.03%). The highest levels of mild hunger were in Al Jawf (47.62%), Al Hudaydah (34.44%), and Al Maharah

governorates. The highest levels of no hunger among conflict migrants were from the Ibb (77.27%), Abyan (73.36%), and Taizz (68.24%) governorates.

Table 2: Level of Hunger across governorates that migrated due to conflict

	No Hunger	Mild Hunger	Moderate Hunger	Severe Hunger
Al Mahwit	39.33%	8.99%	6.74%	44.94%
Dhamar	46.77%	11.29%	12.90%	29.03%
Al Jawf	0.00%	47.62%	23.81%	28.57%
Al Bayda	51.69%	11.02%	9.32%	27.97%
Marib	50.46%	25.23%	4.62%	19.69%
Al Dhale'e	58.82%	13.73%	7.84%	19.61%
Ibb	77.27%	1.82%	3.64%	17.27%
Lahj	63.41%	7.32%	12.20%	17.07%
Aden	53.02%	8.05%	22.15%	16.78%
Amran	38.53%	14.68%	32.11%	14.68%
Sana'a	61.22%	4.08%	20.41%	14.29%
Hajjah	61.17%	6.80%	22.33%	9.71%
Raymah	57.41%	18.52%	15.74%	8.33%
Taizz	68.24%	16.47%	7.65%	7.65%
Al Hudaydah	38.89%	34.44%	21.11%	5.56%
Abyan	73.36%	9.17%	12.01%	5.46%
Al Maharah	57.14%	30.61%	8.16%	4.08%
Sana'a City	91.24%	1.38%	4.61%	2.76%
Shabwah	91.18%	0.00%	7.35%	1.47%
Sa'ada	80.16%	14.49%	4.25%	1.10%
Hadramaut	91.30%	0.00%	8.70%	0.00%
Socotra	100.00%	0.00%	0.00%	0.00%
Pearson Chi2 p-value	< 0.001			

Formatted Table

Table 3 represents the frequency of going without eating for a whole day across governorates that migrated due to conflict. Compared to hunger, the incidence of not eating for an entire day is lower. Conflict migrants in Lahj (19.51%) and Al Jawf (14.29%) had the highest proportion of often going without eating for a whole day. The highest levels of rarely going without eating for an entire day were among conflict migrants in Al Jawf (47.62%), Al Maharah (30.61%), and Al Dhale’e (27.45%).

Table 3: Frequency of going without eating for a whole day across governorates who migrated due to conflict

	Never	Rarely	Sometimes	Often
Lahj	68.29%	4.88%	7.32%	19.51%
Al Jawf	0.00%	47.62%	38.10%	14.29%
Amran	53.21%	24.77%	9.17%	12.84%
Marib	70.15%	14.46%	3.08%	12.31%
Aden	79.87%	1.34%	7.38%	11.41%
Sana'a	73.47%	2.04%	14.29%	10.20%
Taizz	81.76%	7.65%	2.35%	8.24%
Abyan	84.50%	2.18%	8.95%	4.37%
Al Bayda	81.36%	3.39%	11.02%	4.24%
Ibb	94.55%	0.00%	1.82%	3.64%
Al Hudaydah	82.22%	12.22%	2.22%	3.33%
Dhamar	75.81%	19.35%	3.23%	1.61%
Shabwah	94.12%	0.00%	4.41%	1.47%
Sa'ada	88.03%	9.45%	1.42%	1.10%
Al Mahwit	59.55%	12.36%	28.09%	0.00%
Raymah	73.15%	12.04%	14.81%	0.00%
Hajjah	73.79%	11.65%	14.56%	0.00%
Al Dhale'e	58.82%	27.45%	13.73%	0.00%
Hadramaut	91.30%	0.00%	8.70%	0.00%
Al Maharah	61.22%	30.61%	8.16%	0.00%
Sana'a City	97.70%	0.00%	2.30%	0.00%
Socotra	100.00%	0.00%	0.00%	0.00%
Pearson Chi2 p-value	< 0.001			

Formatted Table

Table 4 presents the results of the negative binomial regression, illustrating the impact of experiencing hunger or going without food for a whole day on Yemeni individuals. Individuals who migrated due to conflict significantly increased the incidence of hunger (IRR: 1.262, 95% CI: 1.111–1.435) and reported not eating for the whole day (IRR: 1.251, 95% CI: 1.103–1.516). However, individuals migrating to Yemen had a lower incidence of food insecurity (Hunger: IRR: 0.638, 95% CI: 0.480 – 0.848; Without Eating: IRR: 0.600, 95% CI: 0.367–0.981). Migrating from another governorate did not have a statistically significant impact on the incidence of food insecurity. If the individual migrated due to conflict and from another country, with statistical robustness, it increases the risk of incidence of hunger (IRR: 2.100, 95% CI: 1.279–3.450) by approximately more than two-folds and the likelihood of going without eating (IRR: 6.001, 95%

CI: 2.951–12.206) by about six-folds. Migrating due to conflict from another governorate did not have a statistically significant effect on the incidence of hunger among individuals. Migrating due to conflict from another governorate had a statistically significant impact on the incidence of an individual going without eating for a whole day (IRR: 1.312, p-value < 0.050).

Among the confounding variables, being female is associated with a lower incidence of going hungry (IRR: 0.871, 95% CI: 0.812–0.935) or not eating for a whole day (IRR: 0.782, 95% CI: 0.700–0.873). The education level had a statistically significant effect on decreasing the incidence of hunger (IRR: 0.897, 95% CI: 0.867-0.929) and going without eating for a whole day (IRR: 0.867, 95% CI: 0.821–0.916). Similarly, if the individual is from a wealthier household, there is a significant impact on decreasing the incidence of hunger (IRR: 0.526, 95% CI: 0.491–0.564) and going without eating for a whole day (IRR: 0.436, 95% CI: 0.396–0.481). Individuals living in urban areas are about 2.231- and 3.657-times higher incidence to go hungry (IRR: 2.231, 95% CI: 1.990–2.501) or go without eating for a whole day (IRR: 3.657, 95% CI: 3.086–4.335), respectively.

Table 4: The result of the Negative Binomial Regression

	**Hunger**			**Without Eating**		
	IRR	95% Confidence Interval	P-value	IRR	95% Confidence Interval	P-value
Migration due to conflict	1.262	(1.111, 1.435)	< 0.001	1.251	(1.032, 1.516)	< 0.050
Migrating from another country	0.638	(.480, .848)	<0.010	0.600	(.367, .981)	< 0.050
Migrating from another Governorate	1.003	(.914, 1.099)	NS	0.889	(.761, 1.039)	NS
Migrating due to conflict from another Country	2.100	(1.279, 3.450)	<0.001	6.001	(2.951, 12.206)	< 0.001
Migrating due to conflict from another Governorate	1.014	(.864, 1.188)	NS	1.312	(1.032, 1.667)	< 0.050
**Control variables**						
Female	0.871	(.812, .935)	< 0.001	0.782	(.700, .873)	< 0.001

Formatted Table

Age	0.998	(.996, 1.001)	NS	0.995	(.990, .999)	< 0.010
Household Members	1.002	(.992, 1.012)	NS	0.996	(.981, 1.011)	NS
Education Level	0.897	(.867, .929)	< 0.001	0.867	(.821, .916)	< 0.001
Urban	2.231	(1.990, 2.501)	< 0.001	3.657	(3.086, 4.335)	< 0.001
Composite Wealth Index	0.526	(.491, .564)	< 0.001	0.436	(.396, .481)	< 0.001

IRR: Incidence Rate Ratio (Adjusted for Governorate differences)

Discussion

This research examined how war and conflict-induced migration affected food security in Yemen, utilizing data from the Yemen Multiple Indicator Cluster Survey (MICS) 2022-2023. The investigation unveiled that migration, especially for conflict-related reasons, had a considerable impact on both the prevalence of hunger and the frequency of going without food for an entire day. The results align with the findings of the current literature, which primarily emphasize the detrimental effects of forced migration on food security. It is well-documented that the exodus of populations from their homes and their relocation significantly increases their likelihood of experiencing food insecurity, as they are deprived of access to the means of earning their living, food, and other social services (11).

The findings revealed that people displaced due to conflict experienced a significantly higher likelihood of hunger and skipping meals throughout the day. This thus provides support to the hypothesis that conflict-led displacement reduces food accessibility, deepens economic instability, and intensifies the need for humanitarian aid, which is often insufficient in the majority of the cases (12). The results align with research from other conflict-affected areas, such as Syria and South Sudan, where involuntary migration has been found to contribute to higher food insecurity

due to the collapse of local economies, mass displacement from agricultural land, and the loss of income-generating activities (13).

The data also indicate that the situation of people moving from one country to another due to war or conflict is the most serious problem in food security, with a significantly higher rate of starvation and people not having access to food for a day. This result is consistent with previous studies that showed that international migrants are usually plagued with extra social and economic woes like legal obstacles, discrimination, and exclusion from the social safety net, which underpin their food and nutrition insecurity (8). These issues may also emerge as a result of the struggles migrants face when integrating into communities that are not easily accessible to the host communities, leading to an aggravation of their food insecurity (11).

However, it was found that migration from another part of the governorate within Yemen did not have a statistically significant effect on the incidence of lack of food or going without food for the entire day, which suggests that local displacement within a country may not have an impact as severe as international migration on the food security of a nation. On the contrary, migration due to conflict from a different governorate was statistically significant for the incidence of going without a meal for the whole day, thus showing that even internal displacement can affect access to food, albeit with less impact compared to international migration. These results are consistent with previous literature on food insecurity and migration. Migration driven by conflicts often results in the loss of access to food, which alters social and economic structures and places additional burdens on host communities (11). There is also evidence that these incidences of food insecurity are exacerbated in vulnerable regions, such as Africa, the Middle East, and Eastern Europe (11). Research on forced migrants in Nigeria and Jordan shows that migrants lose their social, cultural, human, and economic capital, which is essential to maintaining food security. It

has also been demonstrated that the influx of migrants into host communities strains both cultural and economic capital within these communities (12,13).

Among the determinants, a few variables were observed to be confounding factors of food insecurity as well. It was noticeable that the female participants were statistically less prone to going to sleep hungry or starving for a whole day. This suggests that women may be the primary figures in eliminating food insecurity in families, as they are typically responsible for household food management (16–18). This result is similar to many other studies that have recognized women as the main authority in handling the food security of their households, especially in areas with constrained resources (19).

The influence of education on food security was also observed, as respondents with higher levels of education had a lower risk of hunger and food insecurity. This finding aligns with earlier research that has emphasized the role of education in promoting economic stability, access to resources, and nutritional knowledge, all of which are key factors in preventing food insecurity (20–23). Educational attainment reduces food insecurity by equipping individuals with literacy skills, nutrition education, and increasing their likelihood of financial stability (19). On the same note, a statistically significant decrease in the ratio of food insecurity was observed in individuals with higher incomes, thus implying the widely observed connection between income and food security (12).

Living in the urban areas led to a higher incidence of hunger and going without food as urban migrants face numerous hurdles in these cities, where the scarcity of resources is severe, and the number of self-sufficiency opportunities in food production is low (24,25). A multitude of studies have indicated that employment issues are the primary cause of stress for urban migrants, and due to the escalating living costs associated with the job, they struggle more to access sufficient food

(26–28). The rural-urban division of food insecurity highlights the importance of understanding the role of the social environment in the interrelationship between migration and food insecurity. Previous literature has portrayed urban sprawl and inefficient city structures in sub-Saharan Africa as leading to socioeconomic segregation, which affects food accessibility for migrants (29).

Finally, the research explored that the wealth index not only had a significant protective effect on food security but also those citizens who were better off in terms of social class on average were the ones who encountered fewer physical sensations of hunger and no experiences of missing a meal. This highlights the importance of financial well-being in achieving food security, especially in conflict-affected areas where access to food is often highly constrained (30).

The research has highlighted that displacements caused by war and conflict are the primary cause of the increased food insecurity in Yemen. The findings of this study have significant policy and humanitarian implications, indicating the necessity of direct aid for conflict migrants and those migrating from abroad, in particular, and suggest addressing the deeper socioeconomic issues that lead to food insecurity in Yemen.

Conclusion and Policy Recommendation

This research presents the insight that the upsurge in hunger among conflict-affected people in Yemen causes significant food crises, especially for displaced individuals who experience greater hunger and are more likely to forgo food for an entire day. The movement of people due to war and conflict presents a challenge not only for the internal inhabitants but also for those crossing borders, which in turn seems to influence the severity of the problem in the latter case. The findings

of this study also indicate that factors such as gender, education, wealth, and the urban-rural location of the population are highly significant in determining food security. According to the findings of this study, women, individuals with higher education, and the affluent were not the minority of people who faced food scarcity; however, urban dwellers showed an increasing vulnerability. This evidence is consistent with the existing literature, which indicates that conflicts can hinder food access, leading to economic instability and necessitating more humanitarian assistance. This research highlights the complex issue of food insecurity in conflict-prone territories, underscoring that migration, particularly forced migration, is a significant contributor to people's exposure to risk of food insecurity. In Yemen, food access interruption due to displacement is accompanied by social and economic discrepancies that, beyond that, nothing else makes even more the difficulties the displaced are facing. It is for these reasons that addressing the issue of hunger among migrants should be a comprehensive process to provide both prompt relief and sustainable solutions simultaneously. Future research should investigate the pathways through which conflict-induced migration affects food insecurity.

Several policy recommendations flow from this research to mitigate the food insecurity faced by the conflict migrants in Yemen. The first and most pressing issue is that humanitarian programs should be boosted, with a focus on the migrant population, especially foreign migrants. These groups face numerous unprecedented challenges that hinder their access to food, stemming from legal, social, and economic inequalities. Thus, the aid program should not only deliver essential food items in an emergency but also explore long-term food security strategies, such as providing nutritional treatments and establishing a sustainable food distribution system in the region.

Second, it is necessary to focus on creating sustainable jobs for the people forced to leave, which is especially important in cities where migrants are at the highest risk. Enabling migrants to be part

of the local economy through job skills training, job market participation, and encouragement of small enterprises can result in better income levels for them and more stable food security. The problem of excessive competition for resources and high living expenses in urban areas should be given special attention owing to the consequences of urban migration. The development of a good transportation system with cities from the periphery might reduce the rate of urban migration in Yemen.

Thirdly, enhancing the availability of education, particularly for women and children, has paramount importance for sustainable food security. Knowledge gained from education enables people to secure decent and stable incomes and access to the better nutrition, and food security. Hence, educational interventions should incorporate elements of both formal education and training in nutrition, agricultural practices, and food security, thereby enabling displaced individuals to possess the necessary information for informed decision-making regarding provision of food. Moreover, assimilating gender-sensitive strategies into food security and migration policies is crucial. Women, children, and old age people have always been the major sufferers within families; therefore, if women are empowered through the provision of resources, education, and economic opportunities, they can substantially contribute to food security within their families, resulting in empowerment through access to land, credit, and entrepreneurial training. This will help them manage food more effectively and assist their families in coping with everyday challenges.

In conclusion, this study reiterates the necessity for comprehensive approaches that address the pressing issue of food security for displaced people in Yemen in a sustainable manner. Humanitarian aid, job provision, education, and gender activities are to be combined into a comprehensive strategy for food security intervention. The realization of these recommendations

can positively influence the resilience of the affected communities, the reduction of food insecurity, and the recovery of Yemen and similar conflict-affected regions in the long run by the governments and humanitarian organizations

Data Availability: Data will be made available on request.

Formatted: Font: 12 pt, Not Bold

References:

1. Watol BS. The Post-2011 Yemen Internationalized Civil War: It's National, Regional and Global Implications. European Journal of Arts, Humanities and Social Sciences. 2024 Jul 1;1(4):3–25.

2. Internal Displacement Monitoring Centre. Global Report on Internal Displacement 2024 [Internet]. Internal Displacement Monitoring Centre; 2024 [cited 2025 May 11]. Available from: https://www.internal-displacement.org/global-report/grid2024

3. Verme P, Schuettler K. The Impact of Forced Displacement on Host Communities. World Bank Policy Research Working Paper (8727) [Internet]. 2019 [cited 2025 May 15]; Available from: https://documents1.worldbank.org/curated/en/654811549389186755/pdf/WPS8727.pdf

4. Becker SO, Ferrara A. Consequences of forced migration: A survey of recent findings. Labour Economics. 2019 Aug;59:1–16.

5. Tandon S, Vishwanath T. The evolution of poor food access over the course of the ~~conflict~~Conflict in Yemen. World Development. 2020 Jun;130:104922.

6. Sihaloho RAH, Prabowo TB, Kusuma RP. Food Crisis in Yemen: The Roles of Food and Agriculture Organization (FAO) from 2015 to 2020. NSJIS. 2022 Jul 23;5(1):59–74.

7. United Nations. UN Yemen Country Results Report 2022. 2022.

8. Al-Waleedi AA, Bin-Ghouth AS. Malnutrition among hospitalized children 12–59 months of age in Abyan and Lahj Governorates / Yemen. BMC Nutr. 2022 Aug 12;8(1):78.

9. Demurtas A, and Occansey Agbeko A. A comparative analysis of two food security and nutrition policies in Yemen between 2018 and 2023: agricultural livelihoods and multisectoral nutrition plan. Global Security: Health, Science and Policy. 2025 Dec 31;10(1):2499453.

10. Dureab F, Al-Falahi E, Ismail O, Al-Marhali L, Al Jawaldeh A, Nuri NN, et al. An Overview on Acute Malnutrition and Food Insecurity among Children during the Conflict in Yemen. Children. 2019 Jun;6(6):77.

11. Iriti M, Vitalini S. Climate change, natural disasters, armed conflicts and migrations at the crossroads between food and nutrition insecurity and undernourishment. FFS. 2025 Jan 2;5(1):1–5.

12. Bertassello L, Winters P, Müller MF. Access to global wheat reserves determines country-level vulnerability to conflict-induced Ukrainian wheat supply disruption. Nat Food. 2023 Aug 3;4(8):673–6.

13. Hassan Onimisi Abu, Megan K Blake. Characterization of food insecurity among the forced migrants in northern Nigeria. Magna Sci Adv Res Rev. 2024 Jan 30;10(1):100–14.

14. Kandeh J, Kumar L. Developing a Relative Ranking of Social Vulnerability of Governorates of Yemen to Humanitarian Crisis. IJGI. 2015 Sep 29;4(4):1913–35.

15. Central Statistical Organization (CSO), United Nations Children's Fund (UNICEF). Yemen multiple Indicator Cluster Survey 2022–2023, Survey findings Report. emen: Central Statistical Organization and United Nations Children's Fund; 2023.

16. Hernández-Moreno A, Gutiérrez-Gutiérrez F, Celedón-Celis N, Girona-Gamarra M, Hochstetter-Diez J. Analysis of Public Policies on Food Security for Older Mapuche Adults in Rural Areas. Foods. 2025 Mar 19;14(6):1055.

17. Hernandez DC. The impact of cumulative family risks on various levels of food insecurity. Social Science Research. 2015 Mar;50:292–302.

18. Hernandez DC, Kim BR, Brooks FP, Gundersen C. The association between hunger-coping economic tradeoffs and food insecurity among female recipients of charitable food assistance. Appetite. 2025 Jan;204:107733.

19. Fitawek W, Hendriks SL. Assessing the impact of inequality on food security. In: Hendriks SL, Babu SC, editors. Handbook on Public Policy and Food Security [Internet]. Edward Elgar Publishing; 2024 [cited 2025 May 15]. p. 260–7. Available from: https://www.elgaronline.com/view/book/9781839105449/book-part-9781839105449-30.xml

20. Tauseef S. The Importance of Nutrition Education in Achieving Food Security and Adequate Nutrition of the Poor: Experimental Evidence from Bangladesh*. Oxf Bull Econ Stat. 2022 Feb;84(1):241–71.

21. Burchi F. Child nutrition in Mozambique in 2003: The role of mother's schooling and nutrition knowledge. Economics & Human Biology. 2010 Dec;8(3):331–45.

22. Burchi F, De Muro P. From food availability to nutritional capabilities: Advancing food security analysis. Food Policy. 2016 Apr;60:10–9.

23. Islam MdS. Do education and health influence economic growth and food security Evidence from Bangladesh. IJHD. 2020;6(1):59.

24. Sohel MdS, Hossen MdS, Hossain B, Sifullah MdK, Galib A. Mapping Out the Food Insecurity of Urban Floating Labour in Mega City of Dhaka: a Qualitative Study. Int Journal of Com WB. 2024 Jun 1;7(2):213–35.

25. Hoque F, Khan MdA, Urme IA, Sultana R, Al Imran S. Food Security Status and Associated Drivers Among Climate Migrant Households in Bangladesh: Insight From Urban Informal Settlements. Food and Energy Security. 2024;13(6):e70020.

26. Wirtz AL, Stevenson M, Guillén JR, Ortiz J, Barriga Talero MÁ, Page KR, et al. Persistent Food Insecurity and Material Hardships: A Latent Class Analysis of Experiences among Venezuelan Refugees and Migrants in Urban Colombia. Nutrients. 2024 Jan;16(7):1060.

27. Borku AW, Utallo AU, Tora TT. The level of food insecurity among urban households in southern Ethiopia: A multi-index-based assessment. Journal of Agriculture and Food Research. 2024 Mar 1;15:101019.

28. Crush J, Frayne ,Bruce, and Pendleton W. The Crisis of Food Insecurity in African Cities. Journal of Hunger & Environmental Nutrition. 2012 Apr 1;7(2–3):271–92.

29. Hemerijckx LM, Janusz K, Van Emelen S, Tumwesigye S, Davis J, Lwasa S, et al. Food accessibility of different socioeconomic groups in sub-Saharan African cities: a mixed-method analysis in Kampala, Uganda. Food Sec. 2022 Jun;14(3):677–94.

30. Arshad A. Impact of financial inclusion on food security: evidence from developing countries. IJSE. 2022 Feb 21;49(3):336–55.

Rebuttal Letter

António Raposo
Academic Editor
PLOS ONE

We are pleased to have received the reviewers' comments on our manuscript entitled: " **From Displacement to Hunger: How Migration Due to Conflict Affects Food Security in Yemen**." The comments were valuable and thoroughly addressed, and the manuscript has been revised accordingly. The modifications (new edition) are highlighted in red in the revised manuscript.

Modifications and explanations in response to the Reviewer's comments are listed below:

Reviewer #1

The study provides robust evidence on the impact of conflict-induced migration on food security in Yemen, with clear policy implications. While limitations exist (e.g., cross-sectional data precluding causal inference on long-term effects, potential underreporting in high-conflict areas), these are common in conflict-region research and do not undermine the core conclusions.

Recommendation: Minor revisions are sufficient for acceptance. Specific suggestions include:

- Expanding the discussion of limitations, such as data collection challenges in unstable regions and self-reporting biases .
    - **Response:** Thank you for your valuable feedback. We have expanded the discussion of limitations to include challenges related to data collection in unstable regions and potential self-reporting biases. These limitations have been addressed in both the methods and discussion sections.

- Enhancing policy recommendations with granularity, e.g., targeting urban migrants and gender-sensitive interventions, given urban residence's strong association with food insecurity (IRR = 2.231) and female protective effects (IRR = 0.871) .
    - **Response:** Thank you for your suggestion. We have enhanced the policy recommendations to include more granularity, specifically targeting urban migrants and gender-sensitive interventions. These adjustments, considering the strong association between urban residence and food insecurity, as well as the protective effects for females, have been reflected in the Discussion, Conclusion, and Policy Recommendation sections.

- Providing sub-group analyses (e.g., migration duration, age cohorts) in supplementary materials to deepen insights into heterogeneity.

- **Response:** Thank you for your valuable feedback. We have incorporated sub-group analyses, such as migration duration and age cohorts, in the annex description to provide deeper insights into heterogeneity.

- The manuscript's methodological rigor, data transparency, and timely relevance to humanitarian crises justify its publication in PLOS ONE following minor refinements.
  - **Response:** Thank you so much for your time and valuable feedback. We appreciate your recognition of the manuscript's methodological rigor, data transparency, and timely relevance. We have addressed the suggested minor refinements and believe the revisions enhance the overall quality of the manuscript.

Reviewer #2

- Ambiguity on unit of analysis: the authors described that 20,000 households were surveyed, but only 13,891 individuals were analyzed.
  - **Response:** Thank you so much for your feedback. We have clarified the unit of analysis in the methods section to address the ambiguity regarding the number of households surveyed and the individuals analyzed.
- The paper is about survey collection data on migration status and food insecurity," but there is no Module/questionnaire names and Specific items or questions used
  - **Response:** Thank you for your feedback. The study utilizes publicly available data from the Yemen Multiple Indicator Cluster Survey (MICS) 2022–2023. We have addressed this issue by providing a detailed explanation of the survey modules, questionnaires, and specific items used in the methodology section.
- Grouped into No hunger, mild hunger. No clear explanation how frequency ranges were mapped into these levels.
  - **Response:** Thank you for this feedback. We have added a clear explanation in the methodology section regarding how the frequency ranges were mapped into the "No hunger" and "Mild hunger" levels. We hope this improves the readability and clarity of the paper.
- Migrating variables 1, 4, and 5 is overlapping. how conflict migration is distinguished across internal and international lines.
  - **Response:** Thank you for this important observation. We have addressed the overlap between migrating variables 1, 4, and 5 and clarified how conflict migration is distinguished across internal and international lines in the revised manuscript.
- Authors mentioned that migration is due to conflict is coded as "1 if yes," but not how this was measured (survey question, self-report, time window).
  - **Response:** Thank you for pointing this out. As mentioned earlier, this study utilized publicly available data from the Yemen Multiple Indicator Cluster Survey (MICS) 2022–2023. We have added a detailed explanation in the methodology section

regarding how migration due to conflict was measured, including the survey question, self-reporting process, and the time window.

- age education and household size are categorical variables but there is no number of categories mentioned similarly for continuous variables is it standardized?
    - **Response:** Thank you for the feedback. We have addressed the categorization of age, education, and household size, as well as the standardization of continuous variables, in the methodology section for better clarity.
- There is inconsistency in P value like 0.050 is used, other times it is written < 0.001 or 0.010
    - **Response:** Thank you for this important feedback. We have corrected the inconsistency in the reporting of p-values and ensured consistent formatting throughout the manuscript.
- IRRs are 2.231 and 3.657, but interpreted too casually as "2 and 4 times
    - **Response:** Thank you so much for your time in reviewing our manuscript and providing detailed feedback. We have revised the interpretation of the IRRs to more accurately reflect their values, rather than the casual "2 and 4 times" phrasing. We believe these changes will significantly improve the quality and readability of the manuscript.

Reviewer #3

1. A major limitation to the current presentation of the manuscript is that causal conclusions are drawn from a cross-sectional survey. Scientifically, it is not possible to draw causal conclusions from surveys that assess a single period of time. I do not doubt the many ways in which conflict-induced migration may impede access to regular meals and food needed to achieve/maintain food security; my concern is solely regarding the cross-sectional nature of the data. I believe this can be easily addressed in the manuscript; causal statements in the introduction, results, and conclusions can be amended to report on associations between conflict-induced migration and food insecurity.
    - **Response:** we have amended the manuscript to replace causal statements with appropriate language reflecting associations between conflict-induced migration and food insecurity, particularly in the introduction and conclusion sections. We appreciate your input and believe these revisions enhance the scientific rigor of our paper.
2. One of the measures used to assess food insecurity in the study assessed 'hunger;' however hunger was not defined, nor were the instructions to respondents and question/response options defined. Based on the context of the study, it sounded like the authors sought to measure hunger over an extended period of time (that was not satisfied by being able to eat). However clearly specifying these details is necessary to understand and interpret the results.

- **Response:** Thank you for pointing this out. We have clarified the definition of "hunger" in the manuscript and provided more detailed instructions for respondents, along with the specific question and response options used in the survey. These revisions should help ensure that the measure is clearly understood and appropriately interpreted in the context of the study.

3. Interpreting the effect size estimates, and moving beyond statements regarding significance/not, would further enhance the manuscript. For example, the findings that migration due to conflict from another country increased risk of hunger more than two-fold (IRR= 2.10) and increased risk of going without food for a day sixfold (IRR=6.001) is staggering and really conveys the extent of the risk to food security when individuals migrate due to conflict from another country. However, as currently presented the magnitude of the risk is not conveyed. I also think it would help to differentiate (or maybe even list first in the tables) migration due to conflict from another country vs migration due to conflict; the latter had effect size estimates that were far lower (~1.25), which is likely because that variable addresses migration due to conflict more broadly. I actually looked at the effect sizes first and was surprised they were not higher; I had to look at the table more closely to realize the variable re: migration due to conflict from another country was really the most prominent one in this case.
    - **Response:** Thank you for your suggestion. We have revised the manuscript to better highlight the effect size estimates, particularly the significant risks associated with migration due to conflict from another country. We have also updated the tables to clearly differentiate this variable from broader migration due to conflict, ensuring the prominence of the more impactful finding is clear.
4. It appears that the authors responded "NA" to the question regarding whether this study was approved by an institutional review board. This caught my attention because the authors report in the descriptive statistics the 22 regions surveyed and their corresponding n's for respondents, some of which are quite small (n=~220). I have concerns that geographic location and responses regarding migration due to conflict could serve to risk identifying some individuals who may have completed the survey, especially if there are few respondents for a region and relatively small numbers of migrants due to conflict from other countries (eg, if ~220 participants completed the survey for a region and there are only ~200 migrants displaced due to conflict from another country).
    - **Response:** Thank you for your concern. As mentioned earlier, this study utilized publicly available data from the Yemen Multiple Indicator Cluster Survey (MICS) 2022–2023. Since the authors did not collect data at the field level, we did not obtain approval from an institutional review board. However, we acknowledge the potential risk of identifying individuals in regions with small sample sizes, and we have taken care to ensure that all data used in the study maintains the privacy and confidentiality of participants.

5. Gender is reported as the label; however sex (male, female) are reported as the response options. Please clarify whether reporting is for gender identify or sex.
   - **Response:** Thank you for pointing this out. We have corrected the manuscript to clarify that the reported data refers to sex (male, female) rather than gender identity. This revision ensures consistency and accuracy in the reporting.
6. Some of the interpretation regarding females and food insecurity, and recommendations for policy solutions, address in a rather factual manner that females are 'the' ones responsible for food management and food security in the household. This is a pretty broad sweeping statement, and more context should be provided to indicate to what degree this is the case for Yemen, as well as the surrounding region (given that the study addresses migration from other countries), and provide citations to support these statements, or amend them accordingly.
   - **Response:** Thank you for your feedback. We have revised the discussion and policy recommendation sections to provide more context regarding the role of females in food management and food security in the household. We have added specific references to the situation in Yemen and the surrounding region to support these statements and avoid overgeneralization. These changes help clarify the degree to which this holds true in the context of the study.

Reviewer #4

1. Some grammatical improvements would improve the text
   - **Response:** We have checked and tried to improve the text quality.
2. The two indicators of food insecurity are simplistic. Explain how they were operationalized (e.g., from which MICS questions)
   - **Response:** Thank you for your suggestion. We have updated the manuscript to clearly explain how the two indicators of food insecurity were operationalized, specifically detailing the MICS questions from which they were derived. This clarification provides a better understanding of how these indicators were measured in the study.
3. Urban residence increasing food insecurity contradicts common assumptions. A brief explanation or hypothesis is warranted (e.g., displaced populations in urban informal settlements).
   Precise confidence intervals (CIs) would make the statistics more informative.
   The statement “general migration decreased food insecurity” needs unpacking. What constitutes “general migration”? Was it internal or international?
   The phrase “worsens food insecurity” implies causality, which is not supportable given the cross-sectional design. Better: “is associated with worsened food
   insecurity.”
   - **Response:** Thank you for your feedback. We have clarified the explanation regarding urban residence and food insecurity, added confidence intervals for the

statistics, defined "general migration" (internal vs. international), and revised the phrasing to "is associated with worsened food insecurity" to align with the cross-sectional design. These changes are now reflected in the introduction, discussion, and conclusion sections.

Regards

Maruf Hasan Rumi
