# Supplementary material for: From displacement to hunger: How migration due to conflict affects food security in Yemen
Source: PLoS One. 2025 Oct 14;20(10):e0333830. doi: 10.1371/journal.pone.0333830 (PMC12520372; doi:10.1371/journal.pone.0333830)
Supplement: Attachment — Submitted filename: PLOS one.docx [file pone.0333830.s002.docx]

**Suggestions**

The paper is about “From Displacement to Hunger: How Migration Due to Conflict Affects Food Security in Yemen” The study addresses an important topic but the study has some methodological and reporting issue. However, authors can incorporate suggestion for better understanding.

**Introduction**

- Introduction: The study objectives is mostly written in future tense.
- Ambiguity on unit of analysis: the authors described that 20,000 households were surveyed, but only 13,891 individuals were analyzed.
- The paper is about survey collection data on migration status and food insecurity," but there is no Module/questionnaire names and Specific items or questions used
- Grouped into No hunger, mild hunger. No clear explanation how frequency ranges were mapped into these levels.
- Migrating variables 1, 4, and 5 is overlapping. how conflict migration is distinguished across internal and international lines.
- Authors mentioned that migration is due to conflict is coded as “1 if yes,” but not how this was measured (survey question, self-report, time window).
- age education and household size are categorical variables but there is no number of categories mentioned similarly for continuous variables is it standardized?
- Sample illustration should be made clear
- There is inconsistency in P value like 0.050 is used, other times it is written < 0.001 or 0.010
- IRRs are 2.231 and 3.657, but interpreted too casually as "2 and 4 times
