# Supplementary material for: From displacement to hunger: How migration due to conflict affects food security in Yemen
Source: PLoS One. 2025 Oct 14;20(10):e0333830. doi: 10.1371/journal.pone.0333830 (PMC12520372; doi:10.1371/journal.pone.0333830)
Supplement: Attachment — Submitted filename: Response to Reviewers.docx [file pone.0333830.s005.docx]

**Rebuttal Letter**

António Raposo
Academic Editor
PLOS ONE

We are pleased to have received the reviewers’ comments on our manuscript entitled: " **From Displacement to Hunger: How Migration Due to Conflict Affects Food Security in Yemen**." The comments were valuable and thoroughly addressed, and the manuscript has been revised accordingly. The modifications (new edition) are highlighted in red in the revised manuscript.

Modifications and explanations in response to the Reviewer’s comments are listed below:

**Reviewer #1**

The study provides robust evidence on the impact of conflict-induced migration on food security in Yemen, with clear policy implications. While limitations exist (e.g., cross-sectional data precluding causal inference on long-term effects, potential underreporting in high-conflict areas), these are common in conflict-region research and do not undermine the core conclusions.

Recommendation: Minor revisions are sufficient for acceptance. Specific suggestions include:

- Expanding the discussion of limitations, such as data collection challenges in unstable regions and self-reporting biases .
  - **Response:** Thank you for your valuable feedback. We have expanded the discussion of limitations to include challenges related to data collection in unstable regions and potential self-reporting biases. These limitations have been addressed in both the methods and discussion sections.
- Enhancing policy recommendations with granularity, e.g., targeting urban migrants and gender-sensitive interventions, given urban residence’s strong association with food insecurity (IRR = 2.231) and female protective effects (IRR = 0.871) .
  - **Response:** Thank you for your suggestion. We have enhanced the policy recommendations to include more granularity, specifically targeting urban migrants and gender-sensitive interventions. These adjustments, considering the strong association between urban residence and food insecurity, as well as the protective effects for females, have been reflected in the Discussion, Conclusion, and Policy Recommendation sections.
- Providing sub-group analyses (e.g., migration duration, age cohorts) in supplementary materials to deepen insights into heterogeneity.
  - **Response:** Thank you for your valuable feedback. We have incorporated sub-group analyses, such as migration duration and age cohorts, in the annex description to provide deeper insights into heterogeneity.
- The manuscript’s methodological rigor, data transparency, and timely relevance to humanitarian crises justify its publication in PLOS ONE following minor refinements.
  - **Response:** Thank you so much for your time and valuable feedback. We appreciate your recognition of the manuscript’s methodological rigor, data transparency, and timely relevance. We have addressed the suggested minor refinements and believe the revisions enhance the overall quality of the manuscript.

**Reviewer #2**

- Ambiguity on unit of analysis: the authors described that 20,000 households were surveyed, but only 13,891 individuals were analyzed.
  - **Response:** Thank you so much for your feedback. We have clarified the unit of analysis in the methods section to address the ambiguity regarding the number of households surveyed and the individuals analyzed.
- The paper is about survey collection data on migration status and food insecurity," but there is no Module/questionnaire names and Specific items or questions used
  - **Response:** Thank you for your feedback. The study utilizes publicly available data from the Yemen Multiple Indicator Cluster Survey (MICS) 2022–2023. We have addressed this issue by providing a detailed explanation of the survey modules, questionnaires, and specific items used in the methodology section.
- Grouped into No hunger, mild hunger. No clear explanation how frequency ranges were mapped into these levels.
  - **Response:** Thank you for this feedback. We have added a clear explanation in the methodology section regarding how the frequency ranges were mapped into the "No hunger" and "Mild hunger" levels. We hope this improves the readability and clarity of the paper.
- Migrating variables 1, 4, and 5 is overlapping. how conflict migration is distinguished across internal and international lines.
  - **Response:** Thank you for this important observation. We have addressed the overlap between migrating variables 1, 4, and 5 and clarified how conflict migration is distinguished across internal and international lines in the revised manuscript.
- Authors mentioned that migration is due to conflict is coded as “1 if yes,” but not how this was measured (survey question, self-report, time window).
  - **Response:** Thank you for pointing this out. As mentioned earlier, this study utilized publicly available data from the Yemen Multiple Indicator Cluster Survey (MICS) 2022–2023. We have added a detailed explanation in the methodology section regarding how migration due to conflict was measured, including the survey question, self-reporting process, and the time window.
- age education and household size are categorical variables but there is no number of categories mentioned similarly for continuous variables is it standardized?
  - **Response:** Thank you for the feedback. We have addressed the categorization of age, education, and household size, as well as the standardization of continuous variables, in the methodology section for better clarity.
- There is inconsistency in P value like 0.050 is used, other times it is written < 0.001 or 0.010
  - **Response:** Thank you for this important feedback. We have corrected the inconsistency in the reporting of p-values and ensured consistent formatting throughout the manuscript.
- IRRs are 2.231 and 3.657, but interpreted too casually as "2 and 4 times
  - **Response:** Thank you so much for your time in reviewing our manuscript and providing detailed feedback. We have revised the interpretation of the IRRs to more accurately reflect their values, rather than the casual "2 and 4 times" phrasing. We believe these changes will significantly improve the quality and readability of the manuscript.

Reviewer #3

1. A major limitation to the current presentation of the manuscript is that causal conclusions are drawn from a cross-sectional survey. Scientifically, it is not possible to draw causal conclusions from surveys that assess a single period of time. I do not doubt the many ways in which conflict-induced migration may impede access to regular meals and food needed to achieve/maintain food security; my concern is solely regarding the cross-sectional nature of the data. I believe this can be easily addressed in the manuscript; causal statements in the introduction, results, and conclusions can be amended to report on associations between conflict-induced migration and food insecurity.
   - **Response:** we have amended the manuscript to replace causal statements with appropriate language reflecting associations between conflict-induced migration and food insecurity, particularly in the introduction and conclusion sections. We appreciate your input and believe these revisions enhance the scientific rigor of our paper.
2. One of the measures used to assess food insecurity in the study assessed 'hunger;' however hunger was not defined, nor were the instructions to respondents and question/response options defined. Based on the context of the study, it sounded like the authors sought to measure hunger over an extended period of time (that was not satisfied by being able to eat). However clearly specifying these details is necessary to understand and interpret the results.
   - **Response:** Thank you for pointing this out. We have clarified the definition of "hunger" in the manuscript and provided more detailed instructions for respondents, along with the specific question and response options used in the survey. These revisions should help ensure that the measure is clearly understood and appropriately interpreted in the context of the study.
3. Interpreting the effect size estimates, and moving beyond statements regarding significance/not, would further enhance the manuscript. For example, the findings that migration due to conflict from another country increased risk of hunger more than two-fold (IRR= 2.10) and increased risk of going without food for a day sixfold (IRR=6.001) is staggering and really conveys the extent of the risk to food security when individuals migrate due to conflict from another country. However, as currently presented the magnitude of the risk is not conveyed. I also think it would help to differentiate (or maybe even list first in the tables) migration due to conflict from another country vs migration due to conflict; the latter had effect size estimates that were far lower (~1.25), which is likely because that variable addresses migration due to conflict more broadly. I actually looked at the effect sizes first and was surprised they were not higher; I had to look at the table more closely to realize the variable re: migration due to conflict from another country was really the most prominent one in this case.
   - **Response:** Thank you for your suggestion. We have revised the manuscript to better highlight the effect size estimates, particularly the significant risks associated with migration due to conflict from another country. We have also updated the tables to clearly differentiate this variable from broader migration due to conflict, ensuring the prominence of the more impactful finding is clear.
4. It appears that the authors responded "NA" to the question regarding whether this study was approved by an institutional review board. This caught my attention because the authors report in the descriptive statistics the 22 regions surveyed and their corresponding n's for respondents, some of which are quite small (n=~220). I have concerns that geographic location and responses regarding migration due to conflict could serve to risk identifying some individuals who may have completed the survey, especially if there are few respondents for a region and relatively small numbers of migrants due to conflict from other countries (eg, if ~220 participants completed the survey for a region and there are only ~200 migrants displaced due to conflict from another country).
   - **Response:** Thank you for your concern. As mentioned earlier, this study utilized publicly available data from the Yemen Multiple Indicator Cluster Survey (MICS) 2022–2023. Since the authors did not collect data at the field level, we did not obtain approval from an institutional review board. However, we acknowledge the potential risk of identifying individuals in regions with small sample sizes, and we have taken care to ensure that all data used in the study maintains the privacy and confidentiality of participants.
5. Gender is reported as the label; however sex (male, female) are reported as the response options. Please clarify whether reporting is for gender identify or sex.
   - **Response:** Thank you for pointing this out. We have corrected the manuscript to clarify that the reported data refers to sex (male, female) rather than gender identity. This revision ensures consistency and accuracy in the reporting.
6. Some of the interpretation regarding females and food insecurity, and recommendations for policy solutions, address in a rather factual manner that females are 'the' ones responsible for food management and food security in the household. This is a pretty broad sweeping statement, and more context should be provided to indicate to what degree this is the case for Yemen, as well as the surrounding region (given that the study addresses migration from other countries), and provide citations to support these statements, or amend them accordingly.
   - **Response:** Thank you for your feedback. We have revised the discussion and policy recommendation sections to provide more context regarding the role of females in food management and food security in the household. We have added specific references to the situation in Yemen and the surrounding region to support these statements and avoid overgeneralization. These changes help clarify the degree to which this holds true in the context of the study.

Reviewer #4

1. Some grammatical improvements would improve the text
   - **Response:** We have checked and tried to improve the text quality.
2. The two indicators of food insecurity are simplistic. Explain how they were operationalized (e.g., from which MICS questions)
   - **Response:** Thank you for your suggestion. We have updated the manuscript to clearly explain how the two indicators of food insecurity were operationalized, specifically detailing the MICS questions from which they were derived. This clarification provides a better understanding of how these indicators were measured in the study.
3. Urban residence increasing food insecurity contradicts common assumptions. A brief explanation or hypothesis is warranted (e.g., displaced populations in urban informal settlements).

Precise confidence intervals (CIs) would make the statistics more informative.

The statement “general migration decreased food insecurity” needs unpacking. What constitutes “general migration”? Was it internal or international?

The phrase “worsens food insecurity” implies causality, which is not supportable given the cross-sectional design. Better: “is associated with worsened food

insecurity.”

- **Response:** Thank you for your feedback. We have clarified the explanation regarding urban residence and food insecurity, added confidence intervals for the statistics, defined "general migration" (internal vs. international), and revised the phrasing to "is associated with worsened food insecurity" to align with the cross-sectional design. These changes are now reflected in the introduction, discussion, and conclusion sections.

Regards

Maruf Hasan Rumi
