# Supplementary material for: From displacement to hunger: How migration due to conflict affects food security in Yemen
Source: PLoS One. 2025 Oct 14;20(10):e0333830. doi: 10.1371/journal.pone.0333830 (PMC12520372; doi:10.1371/journal.pone.0333830)
Supplement: Attachment — Submitted filename: Response_to_comments_plos[1].docx [file pone.0333830.s006.docx]

31 August 2025

António Raposo

Academic Editor

PLOS ONE

We are pleased to have the reviewers’ comments on our manuscript entitled: “**From Displacement to Hunger: How Migration Due to Conflict Affects Food Security in Yemen**”. The comments were addressed, and the manuscript has been revised accordingly. The modifications (new edition) are highlighted in red in the revised manuscript.

Comments from the Reviewers: Modifications and explanations in response to the Reviewer’s comments are listed below:

**Reviewer #1:**

**Critical Revisions Required:**

DATA AVAILABILITY (MUST ADDRESS): Revert to the original public repository link (<https://ghdx.healthdata.org/record/yemen-multiple-indicator-cluster-survey-2022-2023>). And "Data available on request" violates PLOS policy.

**Authors Response#** We have amended the manuscript based on the guidelines of reviewer. [Page 22, Line 370-373]

**Minor Suggestions:**

**Subgroup Analyses:**

Briefly highlight key age/cohort findings (e.g., child migrants’ vulnerability) in the main text.

**Authors Response#** Age cohort tables are included in the paper. [See table 4, Page 13-14, Line 205-209]

**Urban Context:**

Strengthen policy linkages (e.g., connect "urban job creation" to Yemen’s National Food Security Strategy).

**Authors Response#** We have added some sentences to strengthen the policy linkage [Page 21, Line 342-348]

**Policy Nuance:**

Specify how "gender-sensitive interventions" (p. 345) align with Yemen’s cultural frameworks.

**Authors Response#** Thank you for this meticulous observation, we have added more detailed explanation in our revised manuscript. [Page 21, Line 359-361]

**Reviewer #2:**

1. The objectives presented in the introduction should be in past tense

**Authors Response#** We have amended the objectives in past tense. [Page 6, Line 89-96]

2. Yemen’s Central Statistical Organization led this comprehensive survey with technical assistance from UNICEF. “Were the authors part of these organizations?”

**Authors Response#** No, we are not part of these organizations.

3. Line 133….is the 1st variable the sum of 4th and 5th one?

**Authors Response#** Yes.

4. Line 183…. Sentence needs to be rephrased.

**Authors Response#** We have rephrased the sentence.

5. Line 187 represents or presents.

**Authors Response#** it should be “present”. We have corrected the term in the manuscript. [Page 11, Line 188]

6. Line 195 reported experience of hunger

**Authors Response#** We have omitted that line.

7. 202, 204, 211 sentences need revision to make those clear.

**Authors Response#** We have revised the lines based on the reviewer’s suggestion.

8. 239 Either this or thus is enough

**Authors Response#** We have revised the lines based on the reviewer’s suggestion.

**Reviewer #3:**

The authors present a revised version of the manuscript, which is in some instances improved. However, the authors did not address some of my most substantive feedback. My detailed comments are below.

1. The largest concern that I mentioned in my previous review that was not adequately addressed in this revision is regarding the use of causal claims and statements from cross-sectional data. The authors indicated in their response to reviews that they addressed this point by removing causal language throughout. However, this is not the case and causal language is used to discuss the findings throughout. I have pointed out specific instances of such language, although the authors should keep in mind that this is not an exhaustive list:

Lines 159-161 in statistical analysis: describes use of "two methods to investigate the impact of conflict migration on incidence of food insecurity." Using the term impact indicates causal and time sequential analyses; this is not as it is a cross-sectional analysis. The statement follows that "descriptive statistical analysis and .... negative binomial regression" are the methods employed, neither of which can support causal conclusions in this capacity.

Lines 207-208 results: "Migrating from another governorate did not have a statistically significant impact on..."

Lines 214-215: "Migrating due to conflict from another governorate had a statistically significant impact on..."

Lines 232-233 discussion: "had a considerable impact on both the prevalence of hunger and the frequency of going without food for an entire day."

Lines 299-300: "The research has highlighted that displacements caused by war and conflict are the primary cause of the increased food insecurity in Yemen."

**Authors Response#** All wordings have been changed.

2. There are also multiple instances in which the writing would benefit from clarity and careful consideration of framing. For example, the entire first paragraph of the discussion (Lines 229-237) is written in a manner that it is assumed that displacement causes food insecurity; however the authors also present this as the premise they were testing in the study. If it is already a foregone conclusion, it seems like doing the study would be unnecessary. In this case, better specification of the general premise that conflict-based displacement can worsen food insecurity, but this association has not been examined among people in Yemen specifically, would better facilitate the discussion intro and framing.

**Authors Response#** We have rearranged the discussion intro and framing following the reviewer’s suggestion. [Page 16, Line 241-246]

3. In the results, some of what is presented in the tables is repeated in the text; please summarize in the text and leave details for tables.

**Authors Response#** We have updated results.

4. Some statements in the results and throughout are awkward and difficult to understand. one example is lines 209-211: "If the individual migrated due to conflict and from another country, with statistical
robustness, it increases the risk of incidence of hunger (IRR: 2.100, 95% CI: 1.279–3.450) by
approximately more than two-folds...." what is statistical robustness in this context and why not instead just report the p value or CI?

5. Some of the methods and results is repetitive and should be streamlined. For example, Methodology section Lines 98-100 report the study purpose stated immediately above in the intro section.

**Authors Response#** We have made necessary update in results section.

6. Some of the language in the methods is imprecise and should be edited to improve clarity and specificity. For example, Line 143 "Additional variables are included to control mediating and moderating variables." There were no mediating or moderating analyses conducted. These variables were only included as covariates and therefore they should be labeled as such.

**Authors Response#** We have made necessary update in the manuscript.

7. Throught the manuscript, the tense switches from reporting in present tense to past tense and back again. Reporting of methods and results, which were conducted in the past, should be reported in past tense.

**Authors Response#** We have made necessary update in methods and results section.

8. In general, it is not necessary to report on statistical associations of all covariates used in an analysis, unless they are also a key focus of the study. in this case, the control variables do not appear to be critical to the study and therefore each one does not need to be interpreted in the text. If the authors do think interpreting these variables is necessary, then the frame and purpose should be modified to incorporate these.

**Authors Response#** We have made necessary update in the manuscript.

9. In my prior comments, I mentioned that gender as a term was used to report sex (female, male). The authors reported they addressed this, but have not. Gender is reported in the abstract and referred to throughout the discussion and results sections.

**Authors Response#** We have made necessary update in discussion and results section.

10. In the manuscript info section, the authors reported that "data will be made available upon request" however in another area state the data are freely available. Please clarify

**Authors Response#** We have amended the manuscript based on the guidelines of reviewer. [Page 22, Line-370-373]

Regards

Maruf Hasan Rumi
